# Immunotherapy for tuberculosis: current landscape, mechanistic insights, and translational perspectives

**DOI:** 10.3389/fimmu.2026.1859046

**Published:** 2026-07-01

**Authors:** Yanlang He, Xiangping Xie, Shuangyan He, Sha Li, Haolin Zhou, Sheng Wei

**Affiliations:** 1 Department of Infectious Disease, Shaoyang Central Hospital, Shaoyang, China; 2 Department of General Medicine, The Second Affiliated Hospital of Wannan Medical College, Wuhu, China; 3 Department of Clinical Medicine, Changsha Medical University, Changsha, China

**Keywords:** adoptive cell transfer, autophagy, host-directed therapy, immune checkpoint, immunotherapy, *Mycobacterium tuberculosis*, vaccines

## Abstract

Tuberculosis (TB), caused by *Mycobacterium tuberculosis* (Mtb), remains the leading cause of death from a single infectious agent worldwide, with 10.7 million new cases and 1.23 million deaths reported globally in 2024. Although conventional chemotherapy cures most drug-susceptible TB, the rising burden of multidrug-resistant TB (MDR-TB), prolonged treatment regimens, and suboptimal patient adherence continue to undermine control efforts. Against this background, immunotherapeutic approaches have gained renewed interest as rational complements to chemotherapy, building on decades of mechanistic work that has mapped host–pathogen interactions at unprecedented resolution. This review integrates recent advances across the immunotherapy landscape, including next-generation vaccines (M72/AS01E, MTBVAC, VPM1002, BNT164 mRNA candidates), host-directed therapies (HDTs) targeting autophagy, metabolic and inflammatory pathways, cytokine-based and antimicrobial peptide strategies, and adoptive cell therapies. We place particular emphasis on the dual, context-dependent roles of immune checkpoints such as PD-1/PD-L1 in TB, where checkpoint blockade can paradoxically trigger reactivation, and on the cellular heterogeneity revealed by single-cell and spatial transcriptomics of the granuloma. Key translational challenges—the absence of reliable correlates of protection, the demand for precision immunomodulation in comorbid populations (HIV, diabetes), and chronic underfunding of TB research—are discussed alongside emerging opportunities offered by mRNA platforms, repurposed drugs, and multi-omics–guided patient stratification. Collectively, immunotherapy is evolving from an adjunctive concept into a strategic pillar of TB control, with the potential to shorten treatment, prevent relapse, and address drug-resistant disease.

## 1 Introduction

Tuberculosis (TB) remains one of the most formidable infectious disease challenges of the 21st century. According to the World Health Organization (WHO) Global Tuberculosis Report 2025, TB is again the leading cause of death from a single infectious agent worldwide. An estimated 10.7 million people developed TB in 2024 (95% uncertainty interval, 10.0–11.5 million) and 1.23 million died, corresponding to a global incidence of 131 per 100,000 and a case-fatality ratio of 11.5%. Approximately 30 high-burden countries account for the majority of cases, with the WHO South-East Asia and African regions remaining most heavily affected (1). Despite a 12% reduction in incidence and a 29% reduction in mortality since 2015, progress falls well short of the End TB Strategy 2025 milestones of 50% and 75% reductions, respectively. The disease disproportionately affects vulnerable populations, including individuals living with HIV (2), type 2 diabetes (3), and chronic undernutrition (4), as well as those receiving immunosuppressive therapy. Drug-resistant TB continues to compound the burden: approximately 400,000 people developed multidrug-resistant or rifampicin-resistant TB (MDR/RR-TB) in 2024, of whom only 42% accessed appropriate second-line treatment (1).

The immunological control of TB has a long history of translational effort, dating back to the development of the Bacillus Calmette-Guérin (BCG) vaccine. BCG, derived by serial passage of *Mycobacterium bovis* by Calmette and Guérin between 1908 and 1921, entered human use in 1921 and has since been administered to more than 2.5 billion individuals worldwide (5, 6). Incorporated into the WHO Expanded Program on Immunization (EPI), BCG confers 60–80% protection against severe childhood TB manifestations such as miliary disease and tuberculous meningitis (7). However, its efficacy against pulmonary TB in adolescents and adults is inconsistent, ranging from 0 to 80% across trials and geographical settings (8), and protection wanes within 10 to 15 years of vaccination (9). Crucially, BCG does not prevent the progression of latent TB infection (LTBI) to active disease (10). These limitations largely account for the continued dominance of TB as a global killer, a century after BCG was first deployed.

The standard first-line chemotherapeutic regimen for drug-susceptible TB—isoniazid, rifampicin, pyrazinamide, and ethambutol for six months—achieves cure rates above 85% when completed (11). Nevertheless, the prolonged treatment duration erodes adherence, incomplete regimens foster drug resistance (12), and the management of MDR-TB requires 9- to 20-month regimens with substantial toxicity and lower success rates (13). Hepatotoxicity, peripheral neuropathy and other adverse drug reactions further complicate therapy (14). These limitations have prompted a renewed focus on adjunctive strategies, particularly those that engage the host immune response.

Advances in TB immunology have progressively clarified the contributions of macrophages, dendritic cells, T cell subsets, natural killer (NK) cells, and pattern recognition receptors such as the Toll-like receptors (TLRs) to anti-mycobacterial defense (15, 16). On this mechanistic foundation, multiple immunotherapeutic strategies have advanced from bench to clinical evaluation. The M72/AS01E candidate vaccine demonstrated approximately 50% efficacy against progression to pulmonary TB in adults with LTBI, representing a landmark achievement (17). Host-directed therapies (HDTs) aim to rewire host immunometabolic pathways to enhance microbial killing, shorten therapy, and mitigate drug resistance (18). The dual role of immune checkpoints in TB has been clarified with both clinical and experimental rigor (19). Additional approaches target autophagy (20), cytokine signaling (21), and antigen-specific T cell responses (22). Because most of these interventions do not act directly on the bacterium, they are intrinsically less vulnerable to the selective pressures that generate drug resistance—an attribute of strategic value in the fight against MDR/XDR-TB.

This review synthesizes current understanding of host immunity to Mtb and the therapeutic strategies that exploit this biology. We first describe the innate and adaptive arms of anti-TB immunity, highlighting the granuloma as an integrative tissue response and dissecting the paradoxical behavior of immune checkpoints during chronic infection. We then evaluate immunotherapeutic modalities, including new-generation vaccines, HDTs, cytokines and antimicrobial peptides, and adoptive cell transfer. Finally, we discuss the principal translational bottlenecks and emerging opportunities that will shape the next decade of TB immunotherapy research.

A recent and complementary review by Lyu et al. (*Mil Med Res*, 2025) focuses on Mtb-induced host cell exhaustion and discusses adoptive cell therapy and immune checkpoint blockade within a Th1/Th2 framework. Our review differs in scope and organization: we map the full host immune circuitry that Mtb subverts—phagosome maturation, xenophagy, cytosolic surveillance, granuloma biology, and T-cell polarization—and align each immunotherapeutic class (HDT, therapeutic vaccines, cytokine-based interventions, immune checkpoint inhibitors, and adoptive cell therapy) with the specific pathway it engages. Checkpoint inhibition and cell therapy are therefore discussed as part of a broader landscape rather than as the primary scaffold. We also draw on WHO 2025 data and, throughout, emphasize the limitations of murine evidence when extrapolating to human disease.

## 2 Immunological mechanisms in tuberculosis

**Figure 1:**
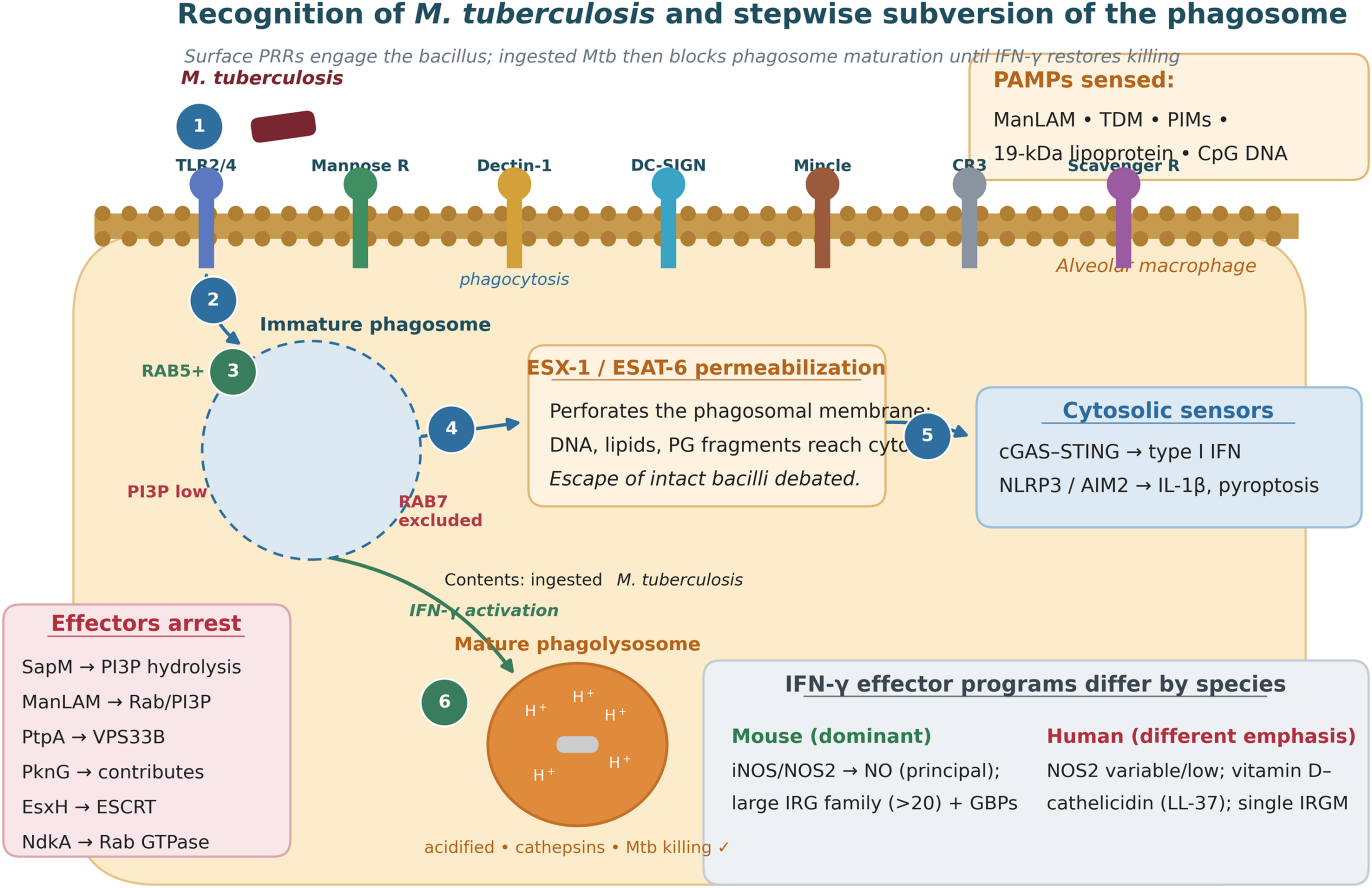
Macrophage recognition of *Mycobacterium tuberculosis* and mechanisms of phagosome subversion. Alveolar macrophages recognize Mtb via TLR2/4, mannose receptor, Dectin-1, DC-SIGN, Mincle, complement receptors, and scavenger receptors, engaging PAMPs including ManLAM, TDM, PIMs, and mycobacterial lipoproteins. Following phagocytosis, Mtb arrests phagosome maturation through a multifactorial program involving SapM (PI3P hydrolysis), ManLAM (PI3P/Rab signaling), PtpA (VPS33B dephosphorylation), PknG (contributes to inhibition; substrates incompletely defined), TDM (membrane composition), EsxH (ESCRT disruption), and NdkA (Rab GTPase signaling). ESX-1/ESAT-6 permeabilize the phagosomal membrane, allowing bacterial molecules (DNA, lipids, peptidoglycan fragments) to engage cytosolic sensors (cGAS–STING, NLRP3, AIM2); full cytosolic escape of intact bacilli remains debated. IFN-γ effector programs are shown separately for murine macrophages (NOS2/NO dominant; large IRG/GBP family) and human macrophages (NOS2 variable; vitamin D–cathelicidin LL-37; single truncated IRGM).

**Figure 2:**
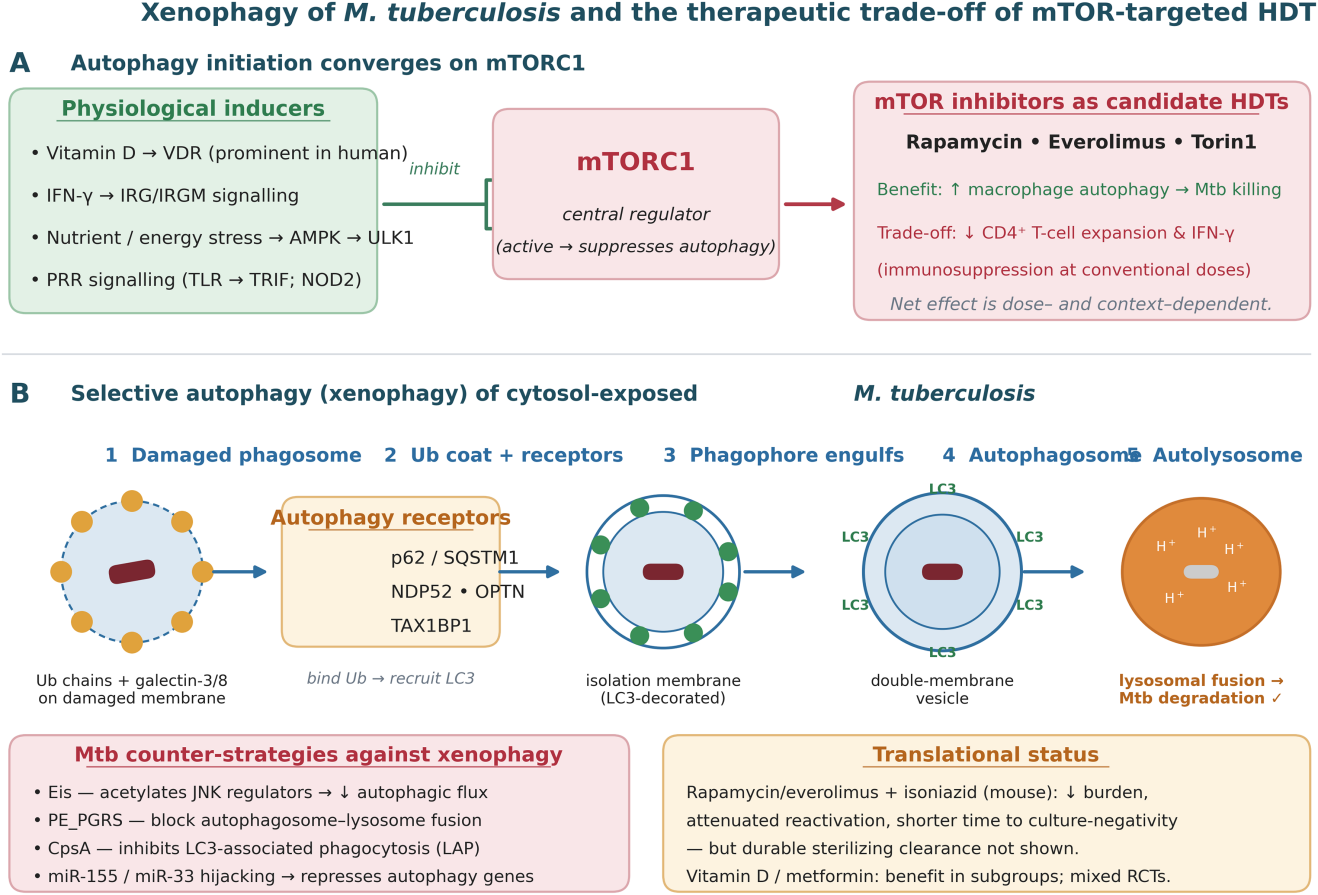
Xenophagy of Mtb and therapeutic trade-offs of mTOR-targeted host-directed therapy. **(A)** Regulation of autophagy initiation: vitamin D–cathelicidin axis (prominent in human), IFN-γ–IRG signaling (extensive in mouse; reduced in human), nutrient/energy stress (AMPK→ULK1), and PRR signaling converge to inhibit mTORC1. mTOR inhibitors (rapamycin, everolimus, Torin1) are candidate HDTs; however, mTOR inhibition also dampens CD4^+^ T-cell expansion and IFN-γ production, an immunosuppressive trade-off that may compromise net protective immunity at conventional doses. **(B)** Selective autophagy (xenophagy): ubiquitin chains accumulate on damaged Mtb-containing phagosomal membranes—and, more variably, on bacterial surface components—recognized by p62/SQSTM1, NDP52, OPTN, and TAX1BP1, which deliver cargo to LC3-decorated autophagosomes for lysosomal degradation. Mtb counter-strategies include Eis acetylation of JNK regulators, PE_PGRS interference with autolysosome fusion, CpsA inhibition of LC3-associated phagocytosis, and host miRNA hijacking (miR-155, miR-33). Translational status: rapamycin/isoniazid combinations in murine models reduce bacterial burden, attenuate reactivation, and shorten time to culture negativity, but durable sterilizing clearance has not been demonstrated.

**Figure 3:**
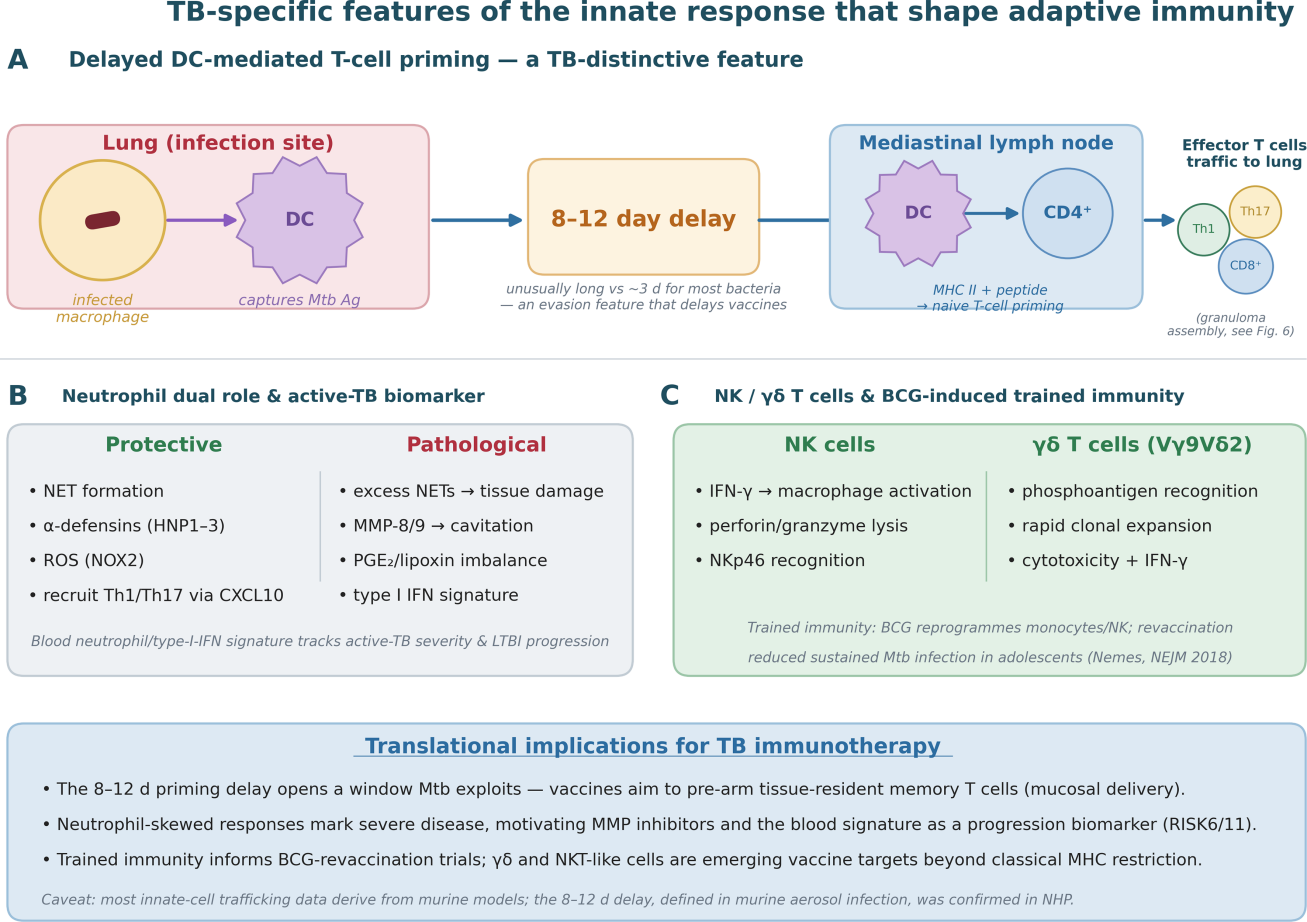
TB-specific features of the innate response that shape adaptive immunity. **(A)** DC-mediated T-cell priming in TB is delayed by 8–12 days—unusually long for a bacterial infection—driven in part by ManLAM/DC-SIGN-mediated suppression of DC maturation; this window allows Mtb to expand unchecked. **(B)** Neutrophil-driven inflammation has a dual role: protective effector functions (NET formation, α-defensin release, ROS) versus pathological amplification (excessive NETs, MMP-8/9–mediated cavitation, type I IFN signature). A neutrophil-driven type I IFN blood transcriptional signature correlates with active TB severity and predicts progression from LTBI (RISK6/RISK11 biomarkers). **(C)** NK cells, γδ T cells (Vγ9Vδ2), and BCG-induced trained immunity in monocytes/NK cells (Kleinnijenhuis 2014, Netea 2020) provide additional innate defense; BCG revaccination reduced sustained Mtb infection in adolescents (Nemes, NEJM 2018). Caveat: most innate-cell trafficking data derive from murine models; the 8–12 day delay was originally defined in murine aerosol infection and confirmed in NHP models.

**Figure 4:**
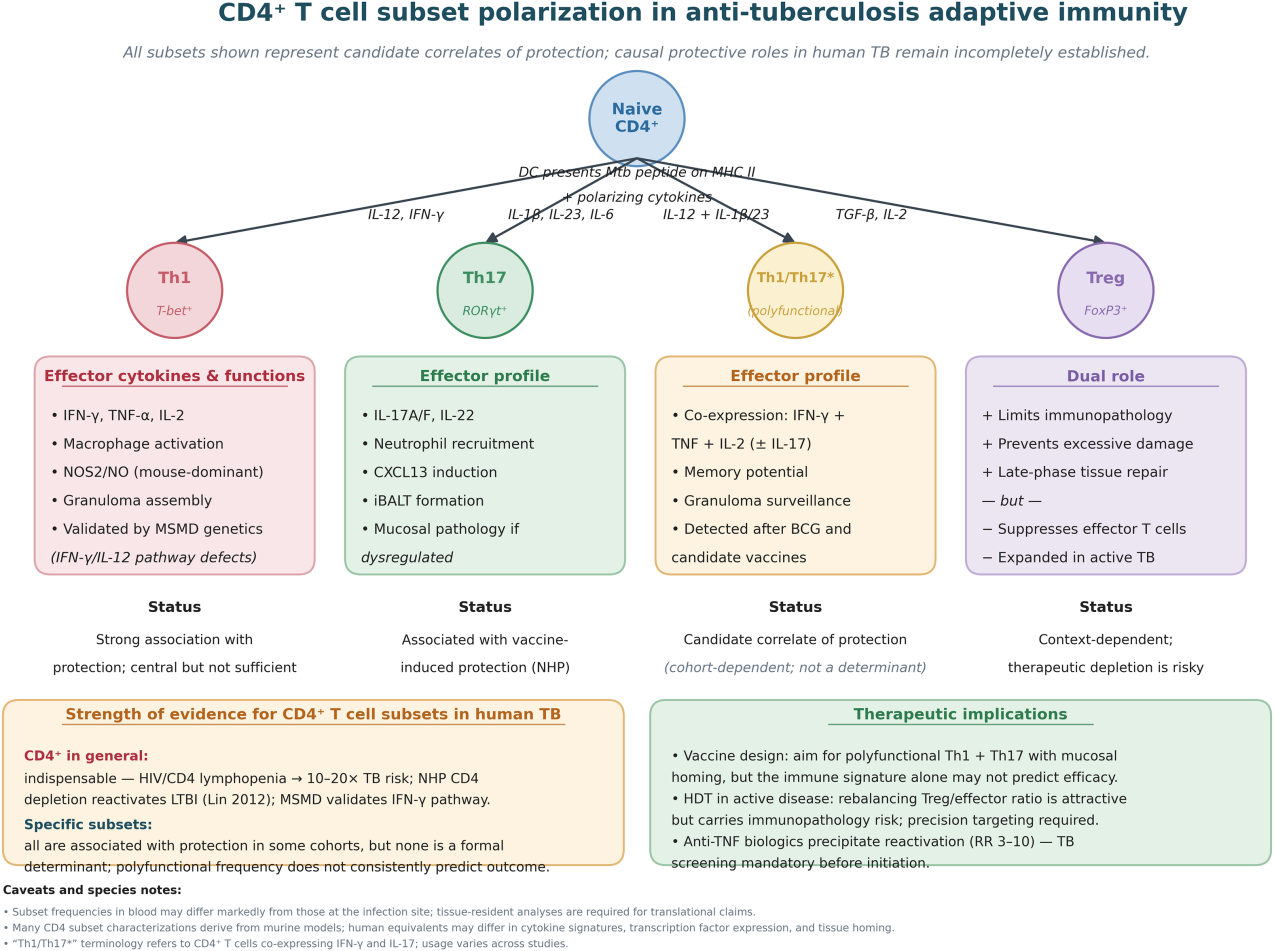
CD4^+^ T cell subset polarization in anti-tuberculosis adaptive immunity. All subsets shown represent candidate correlates of protection; causal protective roles in human TB remain incompletely established. Naive CD4^+^ T cells differentiate under polarizing cytokines into Th1 (T-bet^+^; IFN-γ, TNF-α, IL-2; macrophage activation; validated by Mendelian susceptibility to mycobacterial disease), Th17 (RORγt^+^; IL-17A/F, IL-22; neutrophil recruitment, iBALT formation; associated with vaccine-induced protection in NHP), polyfunctional Th1/Th17* cells (IFN-γ+TNF+IL-2, ± IL-17; candidate correlate, cohort-dependent—not a determinant of protection), and Treg (FoxP3^+^; dual role: limits immunopathology versus suppresses effector responses; context-dependent). CD4^+^ T cells overall are indispensable, as evidenced by HIV-associated TB risk and NHP CD4 depletion reactivating LTBI. Anti-TNF biologics precipitate reactivation (RR 3–10), requiring TB screening before initiation.

**Figure 5:**
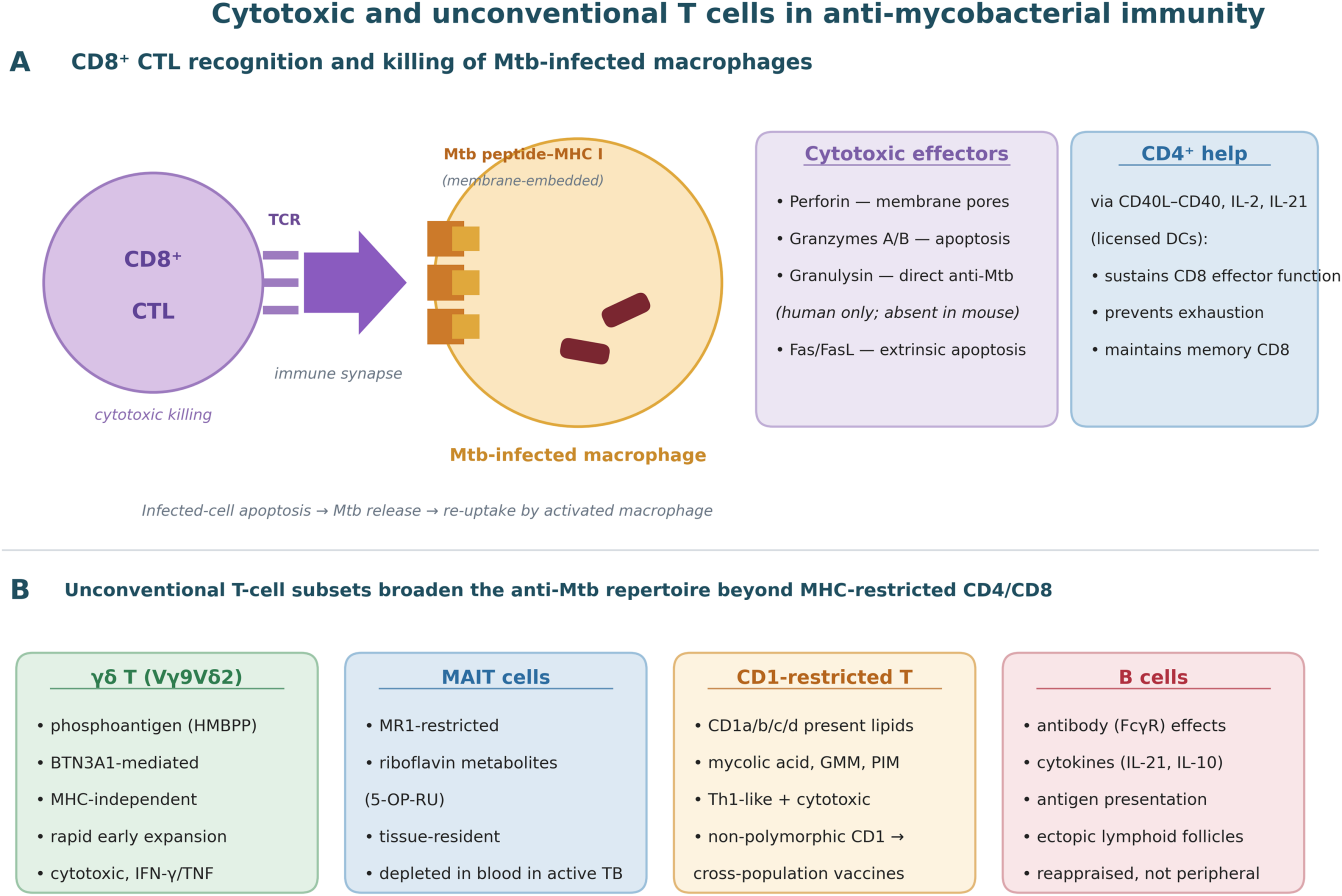
Cytotoxic and unconventional T cells in anti-mycobacterial immunity. **(A)** CD8^+^ CTL killing of Mtb-infected macrophages: TCR recognition of Mtb peptide presented via MHC I triggers release of perforin (membrane pores), granzymes A/B (caspase-3 activation, apoptosis of infected cell), granulysin (direct anti-Mtb activity; human only—absent in mouse), and Fas/FasL extrinsic apoptosis. CD4^+^ T cell help, mediated via CD40L–CD40, IL-2, and IL-21 acting through licensed DCs, sustains CD8^+^ effector function and prevents progressive exhaustion. **(B)** Unconventional T cell subsets broaden the anti-Mtb repertoire beyond MHC-restricted CD4/CD8: γδ T cells (Vγ9Vδ2; phosphoantigen recognition, MHC-independent), MAIT cells (MR1-restricted; recognize Mtb riboflavin metabolites; tissue-resident; depleted in blood during active TB), CD1-restricted T cells (recognize Mtb mycolic acid, sulfoglycolipids, glucose monomycolate; non-polymorphic CD1 favors cross-population vaccine design), and B cells (antibody-mediated effects, cytokine production, antigen presentation, ectopic lymphoid follicles).

**Figure 6:**
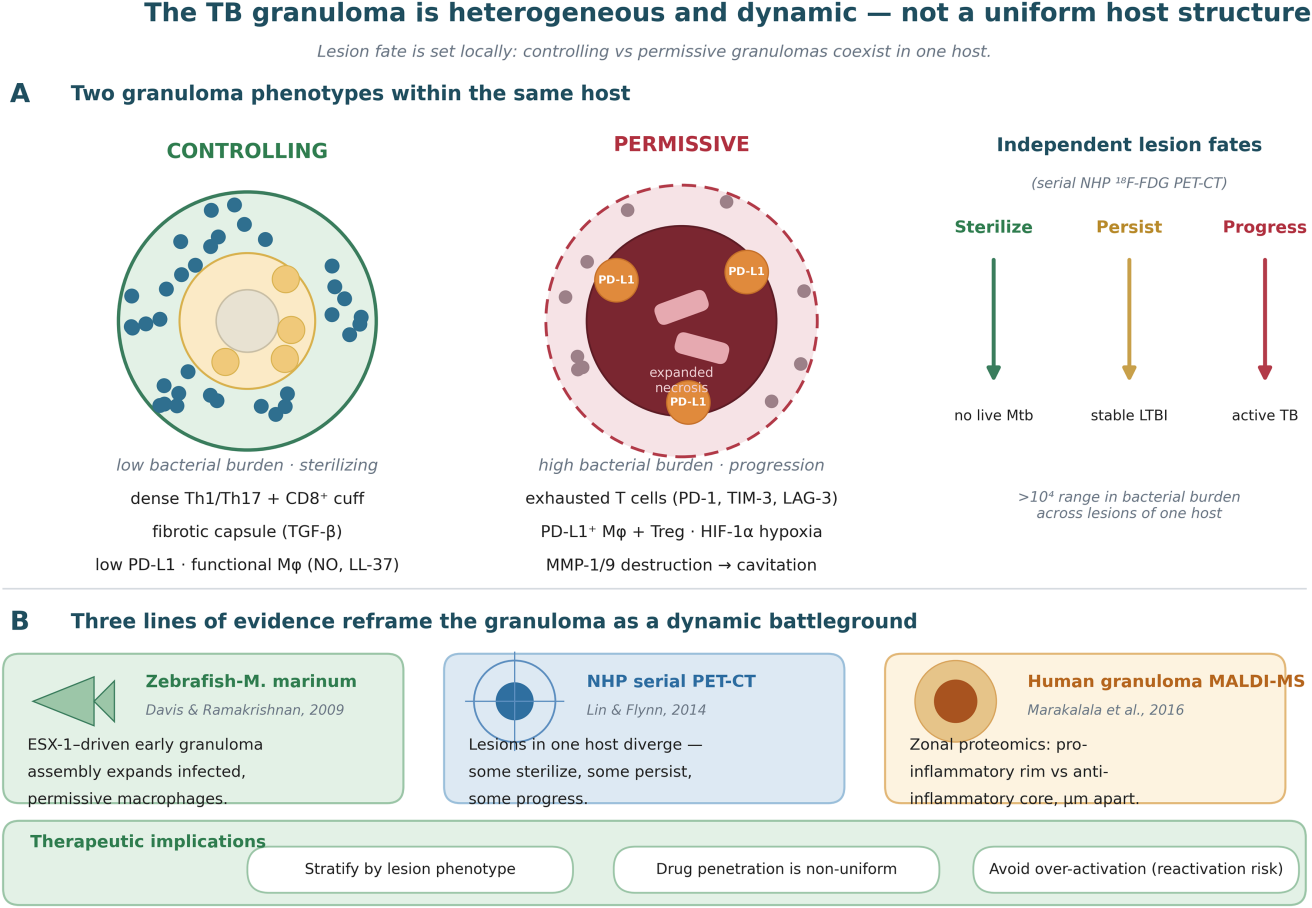
The TB granuloma is heterogeneous and dynamic—not a uniform host structure. **(A)** Side-by-side comparison of controlling versus permissive granulomas within the same host. Controlling granulomas: dense lymphocyte cuffs (Th1/Th17, CD8^+^), small caseous cores, fibrotic encapsulation, functional macrophage effectors, low PD-L1. Permissive granulomas: sparse exhausted lymphocytes (PD-1^+^, TIM-3^+^, LAG-3^+^), expanded caseous necrosis, high PD-L1^+^ macrophages, Treg enrichment, HIF-1α–driven hypoxia, MMP-1/9–mediated tissue destruction. Serial 18F-FDG PET-CT in NHP demonstrates that individual lesions in the same host follow independent trajectories (sterilize/persist/progress), with bacterial burdens differing by >10^4^. **(B)** Three lines of evidence reframe the granuloma: (1) Davis and Ramakrishnan zebrafish–M. marinum model—early granulomas can be exploited by mycobacteria via ESX-1–driven assembly; (2) Lin and Flynn NHP PET-CT—divergent trajectories within one host; (3) Marakalala et al. MALDI-MS spatial proteomics—zonal heterogeneity in human resected granulomas. Therapeutic implications: stratify by lesion phenotype, account for non-uniform drug penetration, avoid over-activation that could disrupt controlling granulomas.

**Figure 7:**
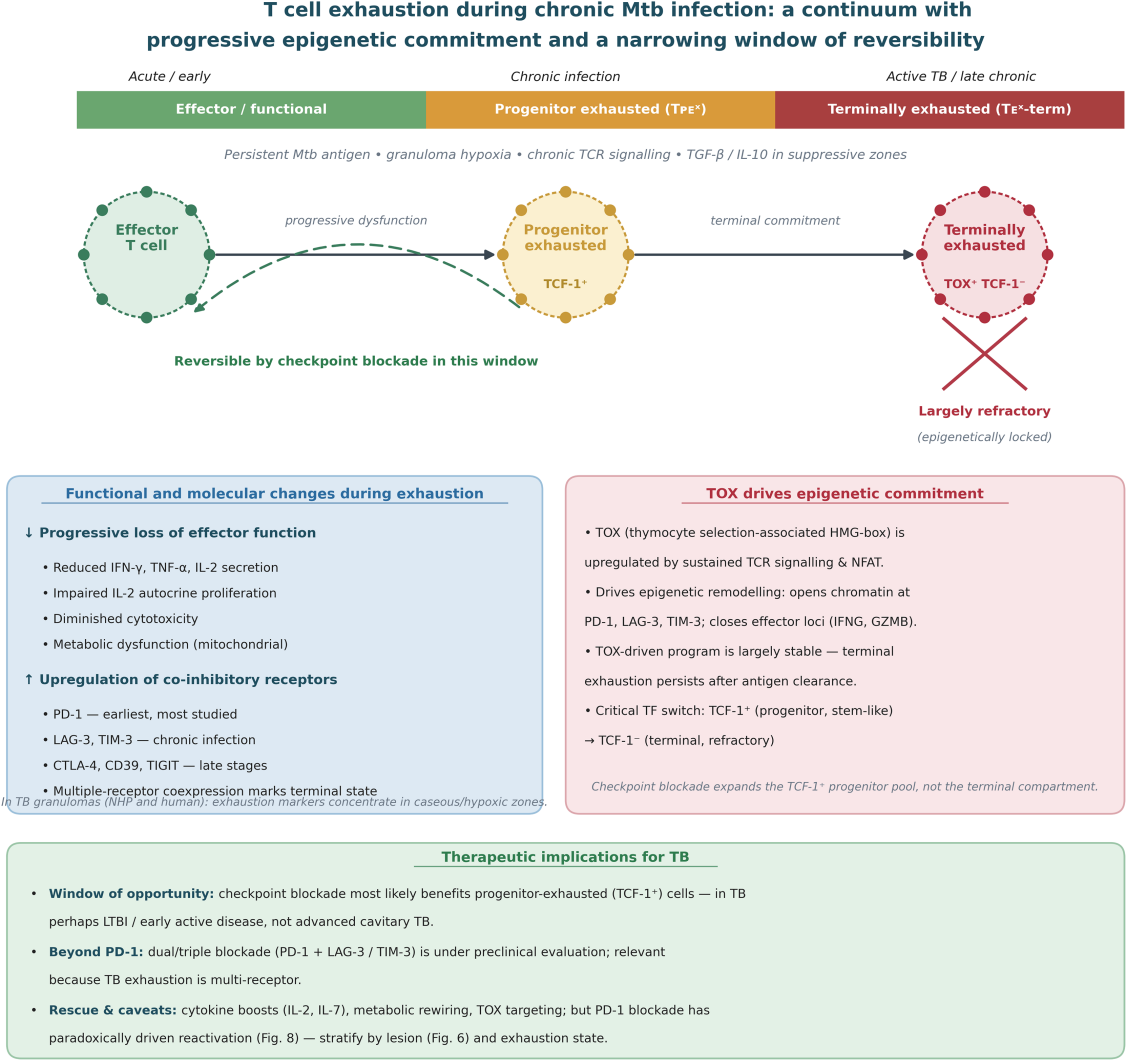
T cell exhaustion during chronic Mtb infection: a continuum with progressive epigenetic commitment and a narrowing window of reversibility. T cells progress from an Effector (functional) state, through a Progenitor exhausted state (TCF-1^+^; stem-like, expandable), to a Terminally exhausted state (TOX^+^ TCF-1^-^; epigenetically committed, largely refractory). Drivers: persistent Mtb antigen, granuloma hypoxia, chronic TCR signaling, and TGF-β/IL-10 in suppressive zones. Functional changes: progressive loss of effector cytokines (IFN-γ, TNF, IL-2), impaired IL-2 autocrine proliferation, diminished cytotoxicity, mitochondrial dysfunction. Inhibitory receptors upregulated in sequence: PD-1 (earliest), LAG-3/TIM-3 (chronic), CTLA-4/CD39/TIGIT (late). TOX (thymocyte selection-associated HMG-box) drives epigenetic remodeling that opens chromatin at PD-1/LAG-3/TIM-3 loci and closes effector loci, locking the terminal state. Checkpoint blockade expands the TCF-1^+^ progenitor pool, not the terminal compartment—defining a narrow rescue window. In TB granulomas (NHP and human), exhaustion markers concentrate in caseous/hypoxic zones.

**Figure 8:**
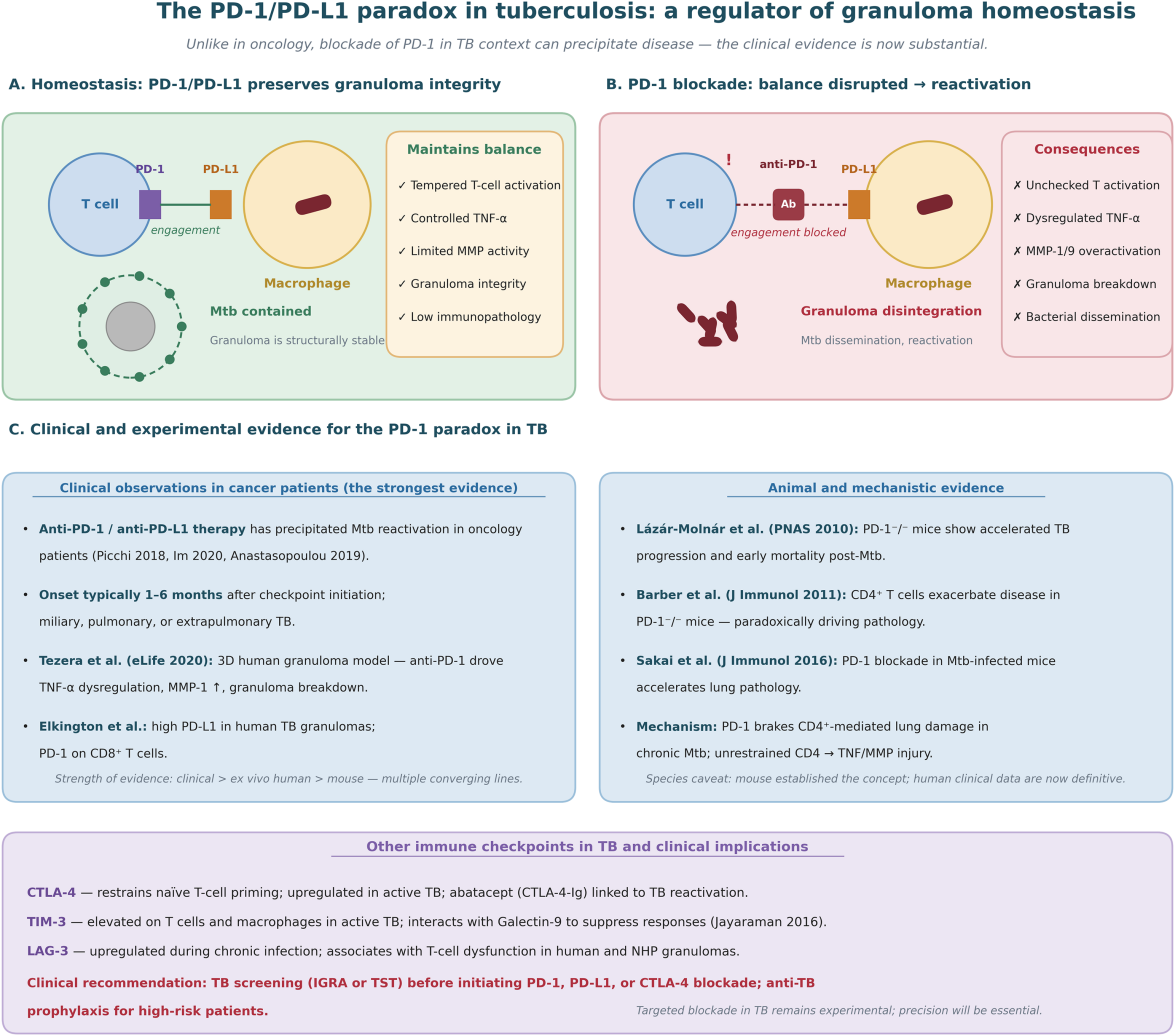
The PD-1/PD-L1 paradox in tuberculosis: a regulator of granuloma homeostasis. Unlike in oncology, PD-1 blockade in the TB context can precipitate disease; clinical evidence is now substantial. **(A)** Homeostasis: PD-1 (T cell)–PD-L1 (macrophage) engagement tempers T-cell activation, controls TNF-α, limits MMP activity, and preserves granuloma integrity. **(B)** PD-1 blockade disrupts this balance: unchecked T activation, dysregulated TNF-α, MMP-1/9 overactivation, granuloma breakdown, bacterial dissemination. **(C)** Clinical evidence—anti-PD-1/PD-L1 therapy in oncology patients has precipitated TB reactivation in multiple case series (Picchi 2018, Im 2020, Anastasopoulou 2019); onset typically 1–6 months after checkpoint initiation. Tezera et al. (eLife 2020) reproduced this in a 3D human granuloma model, with TNF-α dysregulation and MMP-1 elevation driving granuloma breakdown ex vivo. Elkington et al. documented high PD-L1 expression in human TB granulomas. Mouse mechanistic evidence (Lázár-Molnár 2010; Barber 2011; Sakai 2016) established that PD-1 acts as a brake to prevent CD4^+^ T-cell-mediated lung damage during chronic Mtb. Clinical recommendation: TB screening (IGRA or TST) before initiating PD-1/PD-L1/CTLA-4 blockade; anti-TB prophylaxis for high-risk patients.

Effective immunotherapy requires a precise understanding of host defense against Mtb (**Figures 1**–**8**). The anti-mycobacterial response engages innate and adaptive arms of the immune system in an intricately coordinated program, whose complexity exceeds that of most bacterial infections. Mtb has co-evolved a sophisticated arsenal of immune evasion strategies that support long-term intracellular persistence, producing a dynamic equilibrium between host control and pathogen persistence that frames every therapeutic intervention.

### 2.1 Innate immunity

#### 2.1.1 Macrophages: first-line defense and the arena of immune evasion

The innate immune system provides the first line of defense against Mtb. When aerosolized Mtb reaches the alveoli, it is first encountered by alveolar macrophages (AMs), which serve as sentinel phagocytes of the lung. AMs express an extensive repertoire of pattern recognition receptors (PRRs), including Toll-like receptors (TLR2, TLR4, TLR9) (23), C-type lectin receptors such as the mannose receptor, DC-SIGN, Mincle and Dectin-1 (24), complement receptors (CR1, CR3, CR4) (25), and scavenger receptors. These PRRs recognize Mtb-associated pathogen-associated molecular patterns (PAMPs), including lipoarabinomannan (ManLAM), trehalose dimycolate (TDM, also termed cord factor), phosphatidylinositol mannosides (PIMs), and mycobacterial lipoproteins (26), thereby initiating phagocytosis and downstream signaling.

After engulfment, Mtb is sequestered within a phagosome that is normally expected to mature and fuse with lysosomes, creating a degradative phagolysosomal environment. Mtb, however, actively subverts this process through multiple, partially redundant effectors. The secreted acid phosphatase SapM hydrolyzes phosphatidylinositol-3-phosphate (PI3P), one of several mechanisms that impair phagosome maturation (27). Additional Mtb effectors contribute to this block: mannose-capped lipoarabinomannan (ManLAM) interferes with PI3P signaling and Rab GTPase recruitment; the tyrosine phosphatase PtpA dephosphorylates the host vesicular trafficking factor VPS33B; trehalose dimycolate (TDM, cord factor) alters phagosomal membrane composition; EsxH disrupts ESCRT-mediated trafficking; and the nucleoside diphosphate kinase NdkA modulates Rab GTPase signaling. Protein kinase G (PknG), a serine/threonine kinase secreted into the macrophage cytosol, has been proposed to contribute to inhibition of phagosome–lysosome fusion, although its precise host substrates and quantitative contribution remain incompletely defined (28). The ESX-1 type VII secretion system, through effectors such as ESAT-6, permeabilizes the phagosomal membrane, allowing bacterial molecules—including DNA, lipids, and peptidoglycan fragments—to access cytosolic sensors. Full translocation of intact bacilli into the cytosol remains debated and likely occurs only in a subset of infected cells under specific conditions (29). In the cytosol, Mtb-derived DNA is recognized by cGAS–STING, triggering type I interferon (IFN) responses (30). Once activated by IFN-γ, macrophages upregulate inducible nitric oxide synthase (iNOS/NOS2) via the JAK–STAT1 axis, producing nitric oxide (NO) as a principal antimicrobial effector in murine macrophages (31, 32). In human macrophages, by contrast, NOS2 induction is far more variable and generally lower, and bactericidal activity relies more heavily on vitamin D–induced cathelicidin (LL-37), phagolysosomal acidification, and antimicrobial peptides. IFN-γ also stimulates reactive oxygen species (ROS) generation, promotes phagosomal acidification, and enhances lysosomal fusion (33), alongside induction of indoleamine 2,3-dioxygenase (IDO) and immunity-related GTPases (IRGs) (34). Notably, the IRG family is large and functionally important in mice (>20 members, with additional guanylate-binding proteins, GBPs), whereas humans encode only a single truncated IRGM and a reduced GBP repertoire—a major species difference with implications for extrapolating murine findings to human disease.

#### 2.1.2 Autophagy as a critical antimycobacterial pathway

Autophagy has emerged as a pivotal innate defense mechanism in TB. This evolutionarily conserved degradative pathway sequesters cytosolic components, damaged organelles, and invading pathogens within double-membraned autophagosomes that subsequently fuse with lysosomes for content degradation (35). In the context of TB, autophagy performs dual functions: direct clearance of cytosolic Mtb (xenophagy) and modulation of cytokine secretion (20). Ubiquitin chains accumulate principally on damaged Mtb-containing phagosomal membranes and, to a more variable extent, on bacterial surface components. These ubiquitin marks are recognized by selective autophagy receptors including p62/SQSTM1, NDP52, OPTN, and TAX1BP1, which deliver the cargo to LC3-decorated autophagosomes for degradation (36).

The regulation of autophagy centers on the mechanistic target of rapamycin (mTOR): mTOR activation suppresses autophagy, whereas mTOR inhibition induces it (37). On this basis, mTOR inhibitors such as rapamycin and everolimus enhance Mtb killing by macrophages in cultured cells (38). Gutierrez et al. first demonstrated that rapamycin-induced autophagy augments Mtb clearance in human macrophages (39), and subsequent work showed that combined rapamycin and isoniazid treatment reduces bacterial burden, attenuates reactivation, and shortens time to culture negativity in murine models, although durable sterilizing clearance has not been demonstrated and translation to human disease remains to be established (40). Importantly, the systemic immunosuppressive actions of mTOR inhibition—dampened CD4^+^ T cell expansion and IFN-γ production—raise concerns that net protective immunity *in vivo* may be compromised at conventional immunosuppressive doses. Identifying dosing strategies (e.g., pulsed or inhaled regimens) or selective TORC1 modulators that engage autophagy while sparing adaptive immunity is an active area of investigation. Vitamin D enhances antimicrobial activity through a dual mechanism involving cathelicidin (LL-37) induction and autophagy activation (41), providing a molecular rationale for vitamin D as an HDT candidate. IFN-γ similarly promotes autophagic capture of Mtb via the immunity-related GTPase Irgm1 in mice (42). Conversely, Mtb deploys countermeasures—notably the Enhanced Intracellular Survival (Eis) protein, which acetylates autophagy regulators to suppress autophagic flux (43). The ongoing tug-of-war between host autophagy and bacterial evasion strategies offers multiple therapeutic targets for immunomodulatory intervention.

#### 2.1.3 Dendritic cells, neutrophils, and NK cells

Dendritic cells (DCs) bridge innate and adaptive immunity. Pulmonary DCs—including cDC1 and cDC2 subsets—capture and process Mtb antigens before migrating to draining lymph nodes, where they prime naive CD8+ and CD4+ T cells via MHC class I and II antigen presentation, respectively (44). Remarkably, the initiation of adaptive immunity against Mtb is delayed by approximately 8 to 12 days, an unusually prolonged interval compared with most bacterial infections and a feature widely regarded as an immune evasion strategy (45). Mtb infection also impairs DC function: ManLAM binds DC-SIGN and suppresses DC maturation, compromising their capacity to drive T cell proliferation (46).

Neutrophils rapidly accumulate at infection sites and exhibit a dual role in TB (47). On the one hand, they contribute to microbial killing through α-defensin release, neutrophil extracellular traps (NETs), and ROS generation (48). On the other hand, excessive neutrophil recruitment and NET deposition drive tissue injury and inflammatory amplification; notably, a neutrophil-dominated blood transcriptional signature correlates with disease severity in patients with active TB (49).

NK cells contribute to anti-TB immunity via IFN-γ-mediated macrophage activation and direct cytolysis of infected targets through perforin and granzymes (50). The activating receptor NKp46 mediates recognition of Mtb-infected mononuclear phagocytes (51). Recent evidence suggests that NK cells exhibit features of trained immunity, providing heterologous protection against subsequent infections (52). γδ T cells—particularly the Vγ9Vδ2 subset—recognize Mtb-derived phosphoantigens, expand rapidly, and exert cytotoxic effector functions, positioning them as important contributors to early anti-mycobacterial responses (53).

### 2.2 Adaptive immunity

#### 2.2.1 CD4+ T cells: the cornerstone of anti-TB adaptive immunity

Adaptive immunity governs long-term control of Mtb infection. Roughly 2 to 6 weeks after exposure, DCs deliver antigen to draining lymph nodes, where antigen-specific T cells are activated, undergo clonal expansion, and traffic to pulmonary infection foci (44, 45). CD4+ T cells constitute the central pillar of this response. The exceptional susceptibility of HIV-infected individuals to TB disease underscores the indispensability of CD4+ T cells (2), and CD4+ depletion in non-human primate (NHP) models exacerbates acute disease and reactivates LTBI (54).

Th1 cells produce IFN-γ to activate macrophage effector programs, representing the archetypal anti-TB effector mechanism (55). Individuals with Mendelian susceptibility to mycobacterial disease (MSMD)—caused by genetic defects in the IFN-γ pathway—experience severe mycobacterial infections, validating this paradigm in humans (56). Tumor necrosis factor-α (TNF-α) is equally essential for granuloma integrity and containment of Mtb, as evidenced by the elevated reactivation risk among patients receiving anti-TNF-α biologics such as infliximab for autoimmune disorders (57). Th17 cells secrete IL-17, which recruits neutrophils and monocytes to mucosal sites, induces CXCL13 expression, and promotes inducible bronchus-associated lymphoid tissue (iBALT) formation (58). Polyfunctional CD4^+^ T cells co-producing IFN-γ, TNF-α, and IL-2 have been identified in multiple cohorts as correlates of vaccine-induced or BCG-primed responses, although their causal protective role in humans remains unestablished and some studies have found no predictive value for polyfunctional frequency; in active disease, such cells may even associate with bacterial burden rather than with control (59). More recently, a population of CD4^+^ T cells co-expressing IFN-γ and IL-17 (Th1/Th17 or Th1*) has emerged as a candidate correlate of vaccine responses, although the field has moved away from designating any single subset as a definitive determinant of protection (60).

#### 2.2.2 CD8+ T cells and non-conventional T cell subsets

CD8+ T cells mediate cytotoxicity against Mtb-infected cells via perforin/granzyme and Fas/FasL pathways and can directly kill extracellular bacilli through granulysin release (61). Although the independent contribution of CD8+ T cells has been debated, they likely provide critical compensatory protection in contexts of CD4+ dysfunction, such as HIV co-infection (62). Work by Woodworth and colleagues demonstrated that CD4 help is essential to sustain CD8+ effector function and prevent exhaustion during chronic Mtb infection, with unhelped CD8+ cells rapidly losing effector capacity (63).

Non-conventional T cell subsets have gained increasing attention. CD1-restricted T cells recognize Mtb-derived lipid antigens, while MAIT cells—restricted by the MHC class I–related molecule MR1—detect riboflavin metabolites from Mtb. These non-MHC–restricted populations offer novel targets for vaccine and immunotherapeutic design (64). B cells, long considered peripheral to anti-TB immunity, are now recognized for cytokine production, antigen presentation, and contribution to ectopic lymphoid follicles in affected lung tissue (65).

#### 2.2.3 The granuloma as an organized immune response

The granuloma is the pathological hallmark of TB and reflects the host effort to contain Mtb within a structured immunological niche. A typical TB granuloma comprises a central caseous necrotic core, an encircling layer of epithelioid macrophages and multinucleated Langhans giant cells, and an outer cuff of T and B lymphocytes (66). Whether granulomas are predominantly protective or permissive remains under active debate, and recent experimental work supports a more nuanced view than the classical containment paradigm. The long-standing view of granulomas as purely protective structures has been substantially revised. Several lines of evidence challenge this view. First, in the *Mycobacterium marinum*–zebrafish model developed by Davis, Ramakrishnan and colleagues, early granuloma assembly can be actively exploited by mycobacteria—driven by bacterial determinants such as ESX-1—to expand the population of infected, permissive macrophages, with granuloma containment emerging only at later stages (67). Second, serial 18F-FDG PET-CT imaging in cynomolgus macaques (Lin, Flynn and colleagues) has shown that individual granulomas within a single host follow independent trajectories: some sterilize, others persist, and others progress, with bacterial burdens differing by >10^4^ across lesions in the same animal. This argues against any uniform protective or permissive role. Third, spatial proteomic and transcriptomic analyses of human resected granulomas (Marakalala et al.) reveal pronounced zonal heterogeneity in immune cell composition, metabolism, and bacterial localization, with pro-inflammatory rims and anti-inflammatory cores separated by micrometers.

Single-cell RNA sequencing has revealed striking cellular heterogeneity within granulomas. The composition and functional states of immune cells vary markedly across lesions, with direct consequences for bacterial control: “controlling” granulomas suppress Mtb, whereas “permissive” lesions can support bacterial persistence and transmission (68). Spatial transcriptomic studies in NHP models demonstrate pronounced immunosuppressive microenvironments within granulomas, with enrichment of PD-L1+ macrophages and regulatory T cells in specific zones (69). Clinically, granuloma disruption in HIV co-infection or with anti-TNF biologics precipitates TB reactivation, indicating that intact granulomas do exert containment functions in many circumstances. Taken together, the granuloma is best understood not as inherently protective or permissive, but as a dynamic structure whose outcome depends on local immune composition, bacterial burden, and host genetic background—with direct implications for which patients, and which stages of disease, may benefit from specific immunotherapies. This spatial heterogeneity has major therapeutic implications: systemic immune activation may fail to reach granuloma cores, while excessive stimulation risks destabilizing these structures and promoting Mtb dissemination.

#### 2.2.4 T cell exhaustion and immune dysfunction

Persistent antigen stimulation during chronic Mtb infection progressively erodes T cell function—a state termed T cell exhaustion, originally characterized in chronic viral infection and cancer and now well established in TB (70). Exhausted T cells exhibit progressive loss of effector cytokine production (IFN-γ, TNF-α), impaired cytotoxicity, and upregulation of co-inhibitory receptors including PD-1, LAG-3, TIM-3, and CTLA-4 (71). Moguche and colleagues demonstrated in Nature Communications that CD4+ T cells acquire senescent and exhausted phenotypes during chronic Mtb infection, with only a small fraction retaining polyfunctionality within granulomas (72). The hypoxic granuloma microenvironment is thought to accelerate exhaustion, with HIF-1α–driven metabolic reprogramming and co-inhibitory receptor upregulation acting synergistically to impair T cell function (72).

### 2.3 Immune checkpoints and immune tolerance

Immune checkpoints orchestrate the magnitude and duration of anti-mycobacterial responses in complex, context-dependent ways. PD-1 and its ligands PD-L1/PD-L2 represent the most extensively studied axis. In patients with active TB, Mtb-specific CD4+ and CD8+ T cells display elevated PD-1 expression, which correlates with bacterial burden (73). These findings, together with the success of PD-1/PD-L1 blockade in oncology, initially prompted proposals to target this axis as an HDT strategy in TB.

Clinical and experimental evidence, however, has revealed a striking “immune paradox.” Patients receiving PD-1/PD-L1 inhibitors for cancer have experienced reactivation of latent TB, in some cases rapidly after initiation of checkpoint therapy (74). Elkington and colleagues reported characteristic cases of immune checkpoint inhibitor–associated TB reactivation and documented high PD-L1 expression in human TB granulomas alongside co-localization of PD-1 with CD8+ T cells (19). Direct experimental evidence was provided by Lázár-Molnár et al., who demonstrated that PD-1–deficient mice exhibit markedly accelerated disease progression and increased mortality following Mtb challenge (75). Barber and colleagues showed that CD4+ T cells actively exacerbate disease in the absence of PD-1 signaling (76).

These counterintuitive findings indicate that PD-1 does not function simply as an “immune brake” during TB but rather as a regulator that preserves response fidelity, limits inflammation-driven tissue damage, and maintains granuloma homeostasis. Tezera et al. provided a mechanistic explanation, reporting in eLife that PD-1 blockade during TB elicits TNF-α dysregulation, matrix metalloproteinase (MMP) activation, granuloma disintegration, and ultimately bacterial dissemination (77).Other immune checkpoints also modulate TB immunity. CTLA-4 restrains naive T cell priming and effector responses (78), while TIM-3 is highly expressed on T cells and macrophages in active TB and interacts with Galectin-9 to help construct immunosuppressive microenvironments (79). LAG-3 is upregulated during chronic infection and associates with T cell dysfunction (71). Collectively, these observations indicate that the role of immune checkpoints in TB is substantially more complex than in cancer. Blanket checkpoint blockade may not only fail to enhance anti-mycobacterial immunity but also precipitate immunopathology, underscoring the need for precision in any immune checkpoint–directed intervention.

## 3 Immunotherapeutic strategies for tuberculosis

Building on mechanistic insights into TB immunity, investigators have advanced a diverse set of immunotherapeutic strategies designed to complement or enhance conventional chemotherapy. These strategies span vaccine development, host-directed therapy, immunomodulators, and cell-based therapies.

### 3.1 Next-generation vaccines

The limitations of BCG have driven intensive investment in next-generation TB vaccines. According to WHO, 18 candidates were in clinical development as of August 2025—up from 15 in 2024—comprising 4 in Phase I, 8 in Phase II, and 6 in Phase III trials (80). These candidates span preventive and therapeutic indications.

#### 3.1.1 Subunit and adjuvanted vaccines

M72/AS01E is the most advanced TB vaccine candidate currently in clinical evaluation. The vaccine combines two recombinant Mtb fusion proteins (Mtb32A and Mtb39A) with the AS01E adjuvant system (liposomes, monophosphoryl lipid A, and QS-21). In a Phase IIb randomized controlled trial of approximately 3,500 HIV-negative adults with LTBI conducted in Kenya, South Africa, and Zambia, M72/AS01E demonstrated 54.0% efficacy (95% CI, 2.9–78.2) against progression to pulmonary TB after a mean follow-up of 2.3 years (17). The three-year final analysis reported sustained efficacy of 49.7% (95% CI, 2.1–74.2), with an acceptable safety profile and durable immunogenicity: 100% of vaccine recipients seroconverted by month 2, and 99% remained seropositive at month 12 (81). This represents the first clinical demonstration of significant vaccine-mediated protection against progression to active TB in LTBI, a landmark achievement after a century of unmet need. The Phase III registrational trial (NCT06062238), sponsored by the Gates Medical Research Institute, is ongoing in multi-center cohorts including IGRA-positive, IGRA-negative, and HIV-positive participants, with primary completion expected in 2028 (82). A Phase II trial published in Lancet HIV in 2025 confirmed the safety and immunogenicity of M72/AS01E in people living with HIV (83).

Other subunit candidates are advancing through clinical pipelines. H56:IC31, developed by the Statens Serum Institut, combines three Mtb antigens (Ag85B, ESAT-6, and Rv2660c) with the IC31 adjuvant; Phase IIa studies demonstrated acceptable safety and immunogenicity in BCG-vaccinated and LTBI individuals (84). ID93/GLA-SE pairs a four-antigen Mtb fusion protein with a TLR4 agonist adjuvant, and Phase I trials have established its safety profile (85). GamTBvac, a Russian subunit candidate combining Ag85A and an ESAT-6–CFP-10 fusion antigen with a dextran nanoparticle adjuvant, has entered Phase III evaluation (80).

#### 3.1.2 Live vectored and whole-cell vaccines

MTBVAC is a live attenuated vaccine derived from a clinical human Mtb isolate (Mt103) carrying deletions in phoP and fadD26. By retaining ESAT-6 and CFP-10 antigens absent from BCG, MTBVAC is expected to elicit broader T cell responses (86). Early clinical studies in adults and neonates have confirmed safety and immunogenicity, and MTBVAC is now in Phase III evaluation for prevention of Mtb infection in neonates (87). VPM1002, a recombinant BCG expressing listeriolysin O and lacking urease C, is engineered to disrupt phagosomal membranes and enhance MHC class I antigen cross-presentation; it has demonstrated favorable safety in multiple clinical trials (88).

Viral vector platforms leverage attenuated viruses to express Mtb antigens. MVA85A, based on a modified vaccinia Ankara vector expressing Ag85A, did not demonstrate protective efficacy in a Phase IIb trial in South African infants—an outcome that provided important lessons for the field (89). Adenovirus-vectored candidates such as AdHu5Ag85A have exploited intranasal delivery to elicit pulmonary mucosal immunity (90). TB/FLU-05E, a live attenuated influenza virus vector expressing Mtb antigens TB10.4 and HspX, has completed Phase I evaluation in Russia (91).

#### 3.1.3 mRNA vaccines

The clinical success of mRNA vaccine platforms during the COVID-19 pandemic has catalyzed their application to TB. BNT164, developed by BioNTech in partnership with the Bill & Melinda Gates Foundation, is the first TB mRNA vaccine in clinical trials. BNT164 comprises four mRNAs encoding eight Mtb antigens spanning acute, latent, and reactivation phases (Ag85A, ESAT-6, M72, VapB47, Hrp1, RpfA, RpfD, HbhA), delivered via lipid nanoparticles (92). BNT164a1 and BNT164b1 are undergoing Phase Ia dose-escalation studies (NCT05537038, NCT05547464) (93). Preclinical data published in 2025 indicate that both candidates are immunogenic, well tolerated, and efficacious in rodent models, reducing lung Mtb burden across two challenge strains; protection was associated with granuloma infiltration of CD8+ T cells bearing memory precursor phenotypes (94). mRNA platforms offer rapid iterability, flexible manufacturing, and the capacity to encode multiple antigens—advantages tempered by outstanding questions about optimal antigen combinations, durability of cellular immunity, and cold-chain logistics.

#### 3.1.4 Therapeutic vaccines

Therapeutic vaccines aim to enhance immune responses in individuals with active TB or LTBI as an adjunct to chemotherapy. Vaccae™, a heat-killed Mycobacterium vaccae preparation developed in China, is licensed for adjunctive TB therapy. Meta-analyses indicate that Vaccae improves sputum conversion and radiographic resolution when combined with standard chemotherapy (95). RUTI^®^, a liposome-encapsulated fragment preparation of Mtb, elicits polyantigenic responses in LTBI patients after short-course chemoprophylaxis in Phase II trials (96). Mycobacterium indicus pranii (MIP/Immuvac), licensed in India for leprosy, has entered Phase III evaluation as an adjunct in TB (80). DAR-901 (previously SRL-172), an inactivated whole-cell Mtb vaccine, reduced the risk of Mtb infection in BCG-primed individuals in a Phase II trial (97).

### 3.2 Host-directed therapy

HDT has emerged as one of the most translationally promising avenues in TB immunotherapy. By targeting host immunometabolic pathways rather than the bacterium itself, HDTs can enhance antimicrobial activity, reduce immunopathology, and shorten treatment duration (18). Because HDTs do not exert selective pressure on Mtb, they circumvent the development of drug resistance, an attribute particularly valuable in the context of MDR/XDR-TB. Many HDT candidates are repurposed compounds with established safety profiles, favorable cost structures, and broad accessibility.

#### 3.2.1 Metformin

Metformin, a first-line antidiabetic drug, is among the most extensively studied HDT candidates in TB. Its antimycobacterial mechanisms include activation of AMPK, suppression of mTORC1 signaling, induction of autophagy (98), modulation of mitochondrial ROS generation, and attenuation of excessive inflammatory responses (99). Singhal et al. provided the first clinical evidence in Science Translational Medicine, demonstrating across two independent cohorts that metformin augments conventional antitubercular therapy and improves outcomes (100). Multiple retrospective cohort studies reported that metformin reduces the incidence of active TB in patients with type 2 diabetes, with higher doses conferring greater protection (101). Ma et al. documented a decrease in relapse rates among diabetic TB patients receiving metformin over three-year follow-up (102). However, Padmapriydarsini et al. reported mixed results in a randomized controlled trial, with inconsistent effects on sputum culture conversion (103). These divergent findings may reflect differences in patient metabolic status, diabetes control, and Mtb strain characteristics.

#### 3.2.2 Vitamin D

The history of vitamin D in TB therapy extends to the early twentieth-century heliotherapy era. Molecular studies have clarified its mechanisms: 1,25-dihydroxyvitamin D3 binds the vitamin D receptor (VDR) to upregulate cathelicidin (LL-37) and β-defensin 2 expression (41), induce macrophage autophagy (104), promote NO and ROS production, and shape Th1/Th2 balance (105). In a landmark Science paper, Liu et al. elucidated the molecular links between TLR signaling, vitamin D activation, and antimicrobial peptide expression (41). Meta-analyses demonstrate an inverse association between serum 25(OH)D levels and TB incidence (106). However, clinical trials of vitamin D supplementation have yielded inconsistent results: the SUCCINCT study reported accelerated clinical recovery (107), whereas a large RCT by Tukvadze et al. did not detect significant improvements in treatment outcomes (108). Recent meta-analyses suggest that benefits may be restricted to specific subgroups, including patients with severe baseline vitamin D deficiency or certain VDR genotypes (109).

#### 3.2.3 Statins

Beyond their lipid-lowering activity, statins exert immunomodulatory and anti-inflammatory effects. Parihar et al. reported in the Journal of Infectious Diseases that simvastatin reduces Mtb burden in human macrophages and mice by enhancing autophagy and phagosome maturation (110). Because intracellular Mtb depends on host cholesterol metabolism for persistence, as shown by Pandey and Sassetti (111), statin-mediated depletion of cellular cholesterol exerts complementary effects. Ezetimibe similarly limits Mtb survival by lowering intracellular lipid content (112). Drug–drug interactions with rifampicin—a potent CYP3A4 inducer that substantially reduces levels of CYP3A4-metabolized statins (e.g., simvastatin, atorvastatin)—require careful consideration; pravastatin or rosuvastatin, which bypass CYP3A4 metabolism, are preferred where statin–rifampicin co-administration is necessary (113).

#### 3.2.4 Matrix metalloproteinase inhibitors

Excess matrix metalloproteinase (MMP) activity—particularly MMP-1, MMP-3, and MMP-9—drives the tissue destruction and cavitation characteristic of TB (114). Cavity formation not only causes irreversible lung damage but also represents the anatomical precondition for droplet transmission. Doxycycline, a broad-spectrum MMP inhibitor, reduces MMP-1 and MMP-9 expression in Mtb-infected lung tissue, as demonstrated by Walker et al. in AJRCCM (115). The PredDOX Phase II trial (NCT02774993) in South Africa evaluated doxycycline in combination with standard chemotherapy to limit tissue damage. Selective inhibitors targeting specific MMP subtypes are also under preclinical investigation (116).

#### 3.2.5 NSAIDs and corticosteroids

Non-steroidal anti-inflammatory drugs (NSAIDs) modulate TB-associated inflammation by inhibiting cyclooxygenases and reducing prostaglandin E2 (PGE2) synthesis. Ibuprofen has been shown to lower lung bacterial burden and attenuate inflammation in murine TB models (117), and aspirin participates in inflammation resolution through lipoxin synthesis. PGE2 exerts dual effects in TB, maintaining macrophage membrane integrity and supporting early autophagy (118), so the timing and dosing of NSAIDs require careful optimization. Corticosteroids have clear roles in tuberculous meningitis and pericarditis, where dexamethasone significantly reduces mortality (119); however, systemic corticosteroid use in routine pulmonary TB is limited by immunosuppressive risks.

#### 3.2.6 Nutritional supplementation and immunometabolic support

Undernutrition is the largest population-attributable risk factor for tuberculosis worldwide, exceeding HIV and diabetes in absolute terms across most high-burden settings, yet it has historically received far less attention than pharmacological host-directed therapy (4, 144). Protein-energy malnutrition compromises both arms of antimycobacterial immunity, reducing CD4+ T cell numbers and IFN-γ output, blunting macrophage effector function, and lowering circulating leptin and antimicrobial peptide levels—a constellation sometimes described as nutritionally acquired immunodeficiency. Because these deficits are in principle reversible, nutritional repletion constitutes a host-directed intervention that is mechanistically distinct from the immunomodulatory agents discussed above and that acts on the upstream determinants of immune competence.

The strongest clinical evidence derives from the RATIONS cluster-randomized trial in Jharkhand, India, in which monthly food rations provided to household contacts of patients with microbiologically confirmed pulmonary tuberculosis reduced incident disease by 39–48% over two years of follow-up (141). Within the nested patient cohort, nutritional support was associated with improved treatment success, faster recovery of body weight and physical function, and a marked reduction in mortality (142). These results place nutrition among the very few host-directed strategies supported by randomized evidence for both prevention and treatment, and they strengthen the rationale for embedding nutritional assessment and support within routine tuberculosis care, particularly in food-insecure populations. Beyond macronutrient repletion, micronutrients with defined immunological roles—including vitamin D (Section 3.2.2), zinc, vitamin A, and selenium—may confer additional benefit in deficient individuals, although trial findings have been heterogeneous and blanket supplementation is not currently warranted (143). The nutritional dimension therefore complements rather than competes with the pharmacological HDT approaches above and is arguably the most scalable and equitable among them.

### 3.3 Immunomodulators

#### 3.3.1 Cytokine therapy

Given the pivotal role of IFN-γ in anti-TB immunity, recombinant IFN-γ (rIFN-γ) delivered via aerosol was among the earliest immunotherapeutic interventions for drug-resistant TB. Condos et al. reported in Lancet that aerosolized rIFN-γ reduced sputum bacterial burden in MDR-TB (120), although systemic administration is limited by inflammatory toxicity. IL-2 was evaluated as an adjunct to TB chemotherapy, but Johnson et al. failed to detect significant clinical benefit in their RCT (121). Aerosolized GM-CSF has shown potential to enhance alveolar macrophage function and accelerate sputum clearance in selected studies (122). Novel cytokine constructs, including IL-24/IL-12 fusion proteins and recombinant IL-15, are in preclinical development.

#### 3.3.2 Antimicrobial peptides and natural immunomodulators

Antimicrobial peptides (AMPs) serve as key innate immune effectors. LL-37 disrupts Mtb membrane integrity, induces autophagy, and modulates neutrophil and macrophage inflammatory responses (123). Rivas-Santiago et al. documented LL-37 expression in Mtb-infected alveolar macrophages, monocytes, and epithelial cells (123). Defensins—both α-defensins (HNP1-3) and β-defensins (HBD-2, HBD-3)—demonstrate anti-Mtb activity (124). The flavonoid luteolin acts as an immunomodulatory adjunct to isoniazid therapy by enhancing macrophage autophagy and modulating NF-κB and STAT3 signaling, as reported by Singh et al. in PLoS Pathogens (125). L-arginine, the substrate for NO synthesis, has also been shown by McKell et al. to promote anti-Mtb macrophage activity via NO-independent metabolic reprogramming (126). Additional natural products such as all-trans retinoic acid (ATRA), resveratrol, and curcumin exhibit *in vitro* immunomodulatory and anti-Mtb activities (127).

### 3.4 Cell-based immunotherapy

Cell-based immunotherapy represents one of the most forward-looking directions in TB research. Drawing on the success of adoptive cell transfer (ACT) in oncology, investigators are exploring similar strategies in TB. Parida et al. outlined a conceptual framework for T cell therapy in infectious diseases in Clinical Infectious Diseases, proposing that ex vivo–expanded Mtb-specific T cells could be reinfused to enhance bacterial clearance (22). Preclinical studies have demonstrated that adoptive transfer of Mtb-specific CD4+ T cells reduces pulmonary bacterial burden in immunodeficient mice (128).

Whether chimeric antigen receptor (CAR) T cell therapy can be extended to infectious diseases remains an active area of discussion. Unlike cancer, Mtb resides intracellularly within macrophages, so CAR-T cells cannot engage the bacterium directly. T cell receptor (TCR)–engineered T cells targeting Mtb-derived peptides presented by infected macrophage MHC molecules represent a more rational approach and have undergone proof-of-concept evaluation (129).

Regulatory T cells (Tregs) play a substantial negative regulatory role in TB immunity. Multiple studies have shown that Treg frequencies are elevated in blood and disease sites in active TB, and correlate with impaired Mtb-specific effector responses (130). Selective depletion or functional inhibition of site-specific Tregs could, in principle, restore effective immunity, but the risk of autoimmunity and the need for tissue specificity constrain this approach. Mesenchymal stem cell (MSC) therapy—mediated by paracrine immunomodulation and tissue repair—has demonstrated preliminary safety and feasibility as an adjunct in MDR-TB treatment (131).

### 3.5 Summary of immunotherapeutic candidates

**Table 1** summarizes the current landscape of TB immunotherapeutic candidates under clinical or preclinical investigation, detailing drug name, mechanism of action, key supporting studies, and year of publication.

**Table 1:** Candidate agents and vaccines in tuberculosis immunotherapy.

Agent/candidate	Mechanism of action	Key supporting study (reference)	Year
*[I. Preventive vaccines]*			
BCG (Bacillus Calmette–Guérin)	Live attenuated M. bovis; induces Th1 responses; effective in childhood TB only	Mangtani P, et al. Clin Infect Dis (8)	2014
M72/AS01E	Subunit fusion protein (Mtb32A + Mtb39A) with AS01E adjuvant; induces polyfunctional CD4+ T cells	Tait DR, et al. N Engl J Med (Phase IIb) (17, 81)	2018/2019
MTBVAC	Live attenuated M. tuberculosis (phoP/fadD26 double knockout); retains ESAT-6 and CFP-10 epitopes	Tameris M, et al. Lancet Respir Med (87)	2019
VPM1002	Recombinant BCG (expressing listeriolysin O, ΔureC); enhances MHC class I cross-presentation	Nieuwenhuizen NE, et al. Front Immunol (88)	2017
H56:IC31	Three-antigen subunit (Ag85B + ESAT-6 + Rv2660c) with IC31 adjuvant	Luabeya AKK, et al. Vaccine (84)	2015
ID93/GLA-SE	Four-antigen fusion protein with TLR4 agonist adjuvant GLA-SE	Coler RN, et al. Tuberculosis (85)	2018
GamTBvac	Ag85A + ESAT-6–CFP-10 fusion antigen with dextran nanoparticle adjuvant	WHO Global TB Report (80)	2025
MVA85A	Modified vaccinia Ankara vector expressing Ag85A; Phase IIb did not show efficacy	Tameris MD, et al. Lancet (89)	2013
AdHu5Ag85A	Human adenovirus type 5 vector expressing Ag85A; intranasal route drives mucosal immunity	Smaill F, et al. Sci Transl Med (90)	2013
BNT164a1/BNT164b1	mRNA vaccine encoding multiple Mtb antigens (LNP delivery); first TB mRNA candidates in clinic	Vogel N, et al. bioRxiv (92, 94)	2025
TB/FLU-05E	Attenuated influenza virus vector expressing TB10.4 + HspX; intranasal route	ClinicalTrials.gov (91)	2023
*[II. Therapeutic vaccines]*			
Vaccae™	Heat-killed Mycobacterium vaccae; modulates Th1/Th2 balance; accelerates sputum conversion	Yang XY, et al. PLoS One (meta-analysis) (95)	2011
RUTI^®^	Liposome-encapsulated Mtb fragments; therapeutic vaccine for LTBI	Nell AS, et al. PLoS One (96)	2014
MIP/Immuvac	Inactivated Mycobacterium indicus pranii; whole-cell immunomodulatory vaccine	WHO Global TB Report (80)	2025
DAR-901 (SRL-172)	Inactivated non-tuberculous mycobacterial whole-cell vaccine; BCG booster	von Reyn CF, et al. AIDS (97)	2010
*[III. Host-directed therapy (HDT)]*			
Metformin	Activates AMPK; inhibits mTORC1; induces autophagy; modulates mitochondrial ROS; anti-inflammatory	Singhal A, et al. Sci Transl Med (100)	2014
Vitamin D	VDR-mediated cathelicidin (LL-37) induction; autophagy activation; Th1/Th2 modulation	Liu PT, et al. Science (41)	2006
Statins	Enhance autophagy and phagosome maturation; deplete intracellular cholesterol available to Mtb	Parihar SP, et al. J Infect Dis (110)	2014
Ezetimibe	Inhibits cholesterol absorption; limits intracellular lipid supply for Mtb persistence	Pandey AK, Sassetti CM. PNAS (111)	2008
Rapamycin (sirolimus)	mTOR inhibitor; potent autophagy inducer; enhances intracellular Mtb clearance	Gutierrez MG, et al. Cell (20, 39)	2004
Everolimus	mTOR inhibitor; induces autophagy and enhances antigen cross-presentation	Rubinsztein DC, et al. Nat Rev Drug Discov (38)	2012
Doxycycline	Broad-spectrum MMP inhibitor; attenuates lung tissue destruction and cavity formation	Walker NF, et al. Am J Respir Crit Care Med (115)	2012
Ibuprofen	COX inhibitor; reduces PGE2; attenuates inflammation-driven immunopathology	Vilaplana C, et al. J Infect Dis (117)	2013
Dexamethasone	Glucocorticoid; reduces inflammation in tuberculous meningitis and pericarditis	Prasad K, et al. Cochrane Database Syst Rev (119)	2016
N-acetylcysteine (NAC)	Antioxidant; modulates glutathione levels; mitigates oxidative stress	Fatima S, et al. Front Immunol (127)	2021
All-trans retinoic acid (ATRA)	Promotes macrophage autophagy and antigen presentation; regulates T cell differentiation	Fatima S, et al. Front Immunol (127)	2021
*[IV. Cytokines and immunomodulators]*			
Recombinant IFN-γ	Aerosolized delivery; activates macrophage iNOS/NO pathway; enhances microbial killing	Condos R, et al. Lancet (120)	1997
Recombinant IL-2	Promotes T cell proliferation and effector function; modest clinical benefit in RCTs	Johnson JL, et al. Am J Respir Crit Care Med (121)	2003
Recombinant GM-CSF	Aerosolized delivery; enhances alveolar macrophage function	Pedral-Sampaio DB, et al. Braz J Infect Dis (122)	2003
LL-37 (cathelicidin)	Endogenous antimicrobial peptide; direct bactericidal activity, autophagy induction, inflammation regulation	Rivas-Santiago B, et al. Infect Immun (123)	2008
α/β defensins	HNP1–3 and HBD-2/HBD-3; membrane-disruption–based antimicrobial peptides	Gao X, et al. Int Immunopharmacol (124)	2024
Luteolin	Natural flavonoid; enhances autophagy; modulates NF-κB and STAT3 signaling	Singh DK, et al. PLoS Pathog (125)	2021
L-arginine	Substrate for NO synthesis; promotes macrophage metabolic reprogramming	McKell MC, et al. Front Immunol (126)	2021
*[V. Immune checkpoints and cell-based therapies]*			
Anti-PD-1/PD-L1 antibodies	Immune checkpoint blockade (Caution: associated with TB reactivation)	Tezera LB, et al. eLife (77)	2020
Anti-TIM-3 antibodies	Blocks TIM-3/Galectin-9 signaling (preclinical exploration)	Jayaraman P, et al. PLoS Pathog (79)	2016
Mtb-specific T cell therapy	Adoptive transfer of ex vivo–expanded antigen-specific T cells	Parida SK, et al. Clin Infect Dis (22)	2015
TCR-engineered T cells	T cells engineered to recognize MHC-peptide complexes on Mtb-infected cells (proof-of-concept)	Zhuang L, et al. MedComm (129)	2024
Mesenchymal stem cells (MSCs)	Paracrine immunomodulation; tissue repair; adjunctive MDR-TB therapy	Skrahin A, et al. Eur Respir J (131)	2014

Agents listed exert their primary roles in the indicated immune pathways; many repurposed HDT agents (e.g., metformin, statins, vitamin D) are widely used for their original indications (diabetes, hyperlipidemia, etc.) and are listed here only in the context of their mechanistic relevance to TB immunotherapy.

## 4 Discussion: translational challenges and future directions

### 4.1 Immunotherapy for drug-resistant TB

The persistent spread of MDR-TB and extensively drug-resistant TB (XDR-TB) remains among the most daunting challenges in global TB control. WHO estimates approximately 400,000 new MDR/RR-TB cases in 2024, of whom only 42% accessed appropriate second-line treatment (1). Conventional second-line regimens are prolonged, toxic, and achieve only modest cure rates (global MDR-TB treatment success of approximately 68%), and resistance to key second-line agents such as bedaquiline and delamanid is now emerging (132). Because HDT and other immunotherapies do not act directly on Mtb, they are less susceptible to the canonical drug-resistance mechanisms—target mutations, drug efflux, and prodrug-activation defects—that have eroded standard chemotherapy. They are not, however, immune to efficacy erosion: host pathway redundancy, polymorphisms in target pathways, and bacterial adaptation to altered host environments all remain potential routes by which efficacy could be reduced over time. Combining HDT agents—metformin, vitamin D, statins—with novel short-course regimens such as BPaL may augment host antimicrobial activity and partially compensate for reduced antibiotic efficacy against resistant strains (133). Tempering this optimism, however, is the long history of negative or equivocal TB immunotherapy trials (IFN-γ, IL-2, *Mycobacterium vaccae*) and the clear oncology evidence that PD-1 blockade can precipitate TB reactivation. The case for opportunity therefore rests on biomarker-guided patient stratification, host-genotype matching, and combination with short-course drug regimens rather than on broad-population deployment. The potential of therapeutic vaccines to accelerate sputum conversion and reduce relapse rates in patients with drug-resistant disease similarly warrants rigorous evaluation.

### 4.2 The need for correlates of protection

The absence of validated correlates of protection (CoP) remains a critical bottleneck in TB vaccine and immunotherapy development (134). Although IFN-γ production has long served as a surrogate indicator of anti-mycobacterial responses—through assays such as IFN-γ release assays (IGRAs)—accumulating evidence indicates that IFN-γ alone is insufficient to reliably predict protection (135). Candidate CoPs now under investigation include polyfunctional CD4+ responses (59), Th1/Th17* phenotypes (60), tissue-resident memory T cells (TRM) (136), and markers of trained immunity, though none has been fully validated clinically. The biospecimen repository from the M72/AS01E Phase IIb trial represents an invaluable resource for CoP discovery and validation (81).

### 4.3 Precision immunomodulation and individualized therapy

A central challenge for TB immunotherapy lies in achieving precision immune intervention: tailoring interventions to the individual immune phenotype. Patients differ in immune response patterns, comorbid conditions (HIV, diabetes), and genetic background—heterogeneity that makes “one-size-fits-all” strategies suboptimal. High-throughput immune monitoring, single-cell multi-omics (scRNA-seq, CITE-seq) (137), spatial transcriptomics, and artificial intelligence–assisted phenotyping offer promising avenues for precision stratification. The “immune paradox” of PD-1 blockade-associated TB reactivation highlights the importance of precision: indiscriminate immune activation may be ineffective or even deleterious (19, 77).

### 4.4 Translational barriers: funding and infrastructure

Substantial barriers persist in the translational journey from bench to bedside. TB clinical trials are constrained by low event rates, requiring large sample sizes, and by delayed endpoints that mandate prolonged follow-up, yielding high costs for Phase III trials. Advancing a single candidate vaccine from discovery to registration is estimated to cost USD 500 million to 1 billion (138). Global TB research funding reached USD 1.2 billion in 2023, representing only 24% of the WHO-defined target of USD 5 billion per year (80). The 2024 WHO Global TB Report called for increased investment, and the TB Vaccine Accelerator Council, established in 2023, has been tasked with coordinating global stakeholder efforts to accelerate late-stage clinical development (139).

### 4.5 Future directions

Several directions will shape the next decade of TB immunotherapy. The forthcoming Phase III results for M72/AS01E and MTBVAC may yield the first new TB vaccine(s) in a century. mRNA vaccine platforms—exemplified by BNT164—promise rapid iteration, multivalent antigen design, and flexible adaptation to emerging Mtb strains (94). Large-scale RCTs of HDT candidates, particularly metformin and vitamin D, will clarify their positioning as adjuncts to standard chemotherapy. Multi-omics–driven immune phenotyping is poised to enable precision immunomodulation (137), and nanoparticle-based delivery systems may enable targeted pulmonary administration of HDT agents and immunomodulators, enhancing local efficacy while reducing systemic toxicity (140). Finally, rationally designed combinations of vaccines, HDTs, and immunomodulators may produce synergistic benefits, offering pathways toward shortened regimens and improved outcomes in drug-resistant disease.

Prevention through the maintenance of host immune competence is an equally important, and frequently underemphasized, dimension of the immunological approach to tuberculosis. Because progression from infection to active disease is strongly conditioned by the integrity of host defenses, measures that preserve immune function—adequate nutrition, glycemic control in diabetes, correction of vitamin D deficiency, and tuberculosis preventive treatment in high-risk contacts—act in concert with the therapeutic strategies reviewed here to lower the population burden of disease (4, 141). Detailed discussion of these preventive and social determinants is available in dedicated reviews (143).

## 5 Conclusion

TB immunotherapy is entering an era of accelerated progress after more than a century of incremental advance. From the demonstrated efficacy of next-generation vaccines such as M72/AS01E and MTBVAC, to the transformative potential of mRNA platforms; from the mechanistic maturation of HDT candidates including metformin, vitamin D, and statins, to targeted interventions against immunopathology via MMP inhibitors and autophagy modulators; from the nuanced understanding of immune checkpoints such as PD-1/PD-L1 as “balance regulators” rather than simple brakes, to the early exploration of cell-based therapies—immunotherapy is emerging as a diverse strategic pillar alongside conventional chemotherapy.

Key challenges remain, including the identification and validation of correlates of protection, the systematic evaluation of optimal immunotherapy–chemotherapy combinations, the development of precision immunomodulation strategies for patients with drug-resistant TB and comorbid immunodeficiency (HIV, diabetes), the calibrated use of checkpoint modulation to enhance microbial killing without triggering immunopathology, and the sustained financing of TB research and development. As immunological understanding deepens, high-throughput technologies mature, and global investment expands, immunotherapy is expected to play an increasingly pivotal role in TB control—advancing the ambition of the WHO End TB Strategy.

## References

[1] World Health Organization . Global Tuberculosis Report 2025. Geneva: World Health Organization (2025).

[2] Murray CJL Ortblad KF Guinovart C Lim SS Wolock TM Roberts DA . Global, regional, and national incidence, prevalence, and mortality of HIV, tuberculosis, and malaria during 1990-2013: a systematic analysis for the Global Burden of Disease Study 2013. Lancet. (2014) 384:1005–70. doi: 10.1016/S0140-6736(14)60844-8 25059949 PMC4202387

[3] Franco JV Bongaerts B Metzendorf MI Risso A Guo Y Peña Silva L . Diabetes as a risk factor for tuberculosis disease. Cochrane Database Syst Rev. (2024) 8:CD016013. doi: 10.1002/14651858.CD016013.pub2 39177079 PMC11342417

[4] Sinha P Davis J Saag L Wanke C Salgame P Mesick J . Undernutrition and tuberculosis: public health implications. J Infect Dis. (2019) 219:1356–63. doi: 10.1093/infdis/jiy675 30476125 PMC6941617

[5] Li J Lu J Wang G Zhao A Xu M . Past, present and future of Bacillus Calmette-Guérin vaccine use in China. Vaccines (Basel). (2022) 10:1157. doi: 10.3390/vaccines10071157 35891320 PMC9320669

[6] Martín C . The dream of a vaccine against tuberculosis; new vaccines improving or replacing BCG? Eur Respir J. (2005) 26:162–7. doi: 10.1183/09031936.05.00109904 15994403

[7] Trunz BB Fine P Dye C . Effect of BCG vaccination on childhood tuberculous meningitis and miliary tuberculosis worldwide: a meta-analysis and assessment of cost-effectiveness. Lancet. (2006) 367:1173–80. doi: 10.1016/S0140-6736(06)68507-3 16616560

[8] Mangtani P Abubakar I Ariti C Beynon R Pimpin L Fine PE . Protection by BCG vaccine against tuberculosis: a systematic review of randomized controlled trials. Clin Infect Dis. (2014) 58:470–80. doi: 10.1093/cid/cit790 24336911

[9] Negi K Bhaskar A Dwivedi VP . Progressive host-directed strategies to potentiate BCG vaccination against tuberculosis. Front Immunol. (2022) 13:944183. doi: 10.3389/fimmu.2022.944183 35967410 PMC9365942

[10] Bruffaerts N Romano M Denis O Jurion F Huygen K . Increasing the vaccine potential of live M. bovis BCG by coadministration with plasmid DNA encoding a tuberculosis prototype antigen. Vaccines (Basel). (2014) 2:181–95. doi: 10.3390/vaccines2010181 26344474 PMC4494193

[11] World Health Organization . Guidelines for Treatment of Drug-Susceptible Tuberculosis and Patient Care, 2017 Update. Geneva: World Health Organization (2017).

[12] Goossens SN Sampson SL Van Rie A . Mechanisms of drug-induced tolerance in Mycobacterium tuberculosis. Clin Microbiol Rev. (2020) 34:e00141-20. doi: 10.1128/CMR.00141-20 33055230 PMC7566895

[13] World Health Organization . WHO Consolidated Guidelines on Tuberculosis. Module 4: Treatment - Drug-Resistant Tuberculosis Treatment, 2022 Update. Geneva: World Health Organization (2022). 36630546

[14] Ramappa V Aithal GP . Hepatotoxicity related to anti-tuberculosis drugs: mechanisms and management. J Clin Exp Hepatol. (2013) 3:37–49. doi: 10.1016/j.jceh.2012.12.001 25755470 PMC3940184

[15] Kaufmann SHE . How can immunology contribute to the control of tuberculosis? Nat Rev Immunol. (2001) 1:20–30. doi: 10.1038/35095558 11905811

[16] Philips JA Ernst JD . Tuberculosis pathogenesis and immunity. Annu Rev Pathol. (2012) 7:353–84. doi: 10.1146/annurev-pathol-011811-132458 22054143

[17] Tait DR Hatherill M Van Der Meeren O Ginsberg AM Van Brakel E Salaun B . Final analysis of a trial of M72/AS01E vaccine to prevent tuberculosis. N Engl J Med. (2019) 381:2429–39. doi: 10.1056/NEJMoa1909953 31661198

[18] Kaufmann SHE Dorhoi A Hotchkiss RS Bartenschlager R . Host-directed therapies for bacterial and viral infections. Nat Rev Drug Discov. (2018) 17:35–56. doi: 10.1038/nrd.2017.162 28935918 PMC7097079

[19] Elkington PT Bateman AC Thomas GJ Ottensmeier CH . Implications of tuberculosis reactivation after immune checkpoint inhibition. Am J Respir Crit Care Med. (2018) 198:1451–3. doi: 10.1164/rccm.201807-1250LE 30141960 PMC6290953

[20] Gutierrez MG Master SS Singh SB Taylor GA Colombo MI Deretic V . Autophagy is a defense mechanism inhibiting BCG and Mycobacterium tuberculosis survival in infected macrophages. Cell. (2004) 119:753–66. doi: 10.1016/j.cell.2004.11.038 15607973

[21] Condos R Rom WN Schluger NW . Treatment of multidrug-resistant pulmonary tuberculosis with interferon-gamma via aerosol. Lancet. (1997) 349:1513–5. doi: 10.1016/S0140-6736(96)12273-X 9167461

[22] Parida SK Poiret T Zhenjiang L Meng Q Heyckendorf J Lange C . T-cell therapy: options for infectious diseases. Clin Infect Dis. (2015) 61:S217–24. doi: 10.1093/cid/civ615 26409284 PMC4583575

[23] Varshney D Singh SV Mohanty KK Tandon B Tyagi P Kumari K . Toll-like receptor 2 (-196 to -174) del and TLR1 743 A>G gene polymorphism-a possible association with drug-resistant tuberculosis in the north Indian population. Front Microbiol. (2024) 14:1305974. doi: 10.3389/fmicb.2023.1305974 38481606 PMC10936010

[24] Tailleux L Schwartz O Herrmann JL Pivert E Jackson M Amara A . DC-SIGN is the major Mycobacterium tuberculosis receptor on human dendritic cells. J Exp Med. (2003) 197:121–7. doi: 10.1084/jem.20021468 12515819 PMC2193794

[25] Schlesinger LS Bellinger-Kawahara CG Payne NR Horwitz MA . Phagocytosis of Mycobacterium tuberculosis is mediated by human monocyte complement receptors and complement component C3. J Immunol. (1990) 144:2771–80. doi: 10.4049/jimmunol.144.7.2771 2108212

[26] Silva Miranda M Breiman A Allain S Deknuydt F Altare F . The tuberculous granuloma: an unsuccessful host defence mechanism providing a safety shelter for the bacteria? Clin Dev Immunol. (2012) 2012:139127. doi: 10.1155/2012/139127 22811737 PMC3395138

[27] Vergne I Chua J Lee HH Lucas M Belisle J Deretic V . Mechanism of phagolysosome biogenesis block by viable Mycobacterium tuberculosis. Proc Natl Acad Sci USA. (2005) 102:4033–8. doi: 10.1073/pnas.0409716102 15753315 PMC554822

[28] Walburger A Koul A Ferrari G Nguyen L Prescianotto-Baschong C Huygen K . Protein kinase G from pathogenic mycobacteria promotes survival within macrophages. Science. (2004) 304:1800–4. doi: 10.1126/science.1099384 15155913

[29] Chandra P Grigsby SJ Philips JA . Immune evasion and provocation by Mycobacterium tuberculosis. Nat Rev Microbiol. (2022) 20:750–66. doi: 10.1038/s41579-022-00763-4 35879556 PMC9310001

[30] Watson RO Bell SL MacDuff DA Kimmey JM Diner EJ Olivas J . The cytosolic sensor cGAS detects Mycobacterium tuberculosis DNA to induce type I interferons and activate autophagy. Cell Host Microbe. (2015) 17:811–9. doi: 10.1016/j.chom.2015.05.004 26048136 PMC4466081

[31] MacMicking JD North RJ LaCourse R Mudgett JS Shah SK Nathan CF . Identification of nitric oxide synthase as a protective locus against tuberculosis. Proc Natl Acad Sci USA. (1997) 94:5243–8. doi: 10.1073/pnas.94.10.5243 9144222 PMC24663

[32] Choi HG Kwon KW Choi S Back YW Park HS Kang SM . Antigen-specific IFN-γ/IL-17-co-producing CD4+ T cells are the determinants for protective efficacy of tuberculosis subunit vaccine. Vaccines (Basel). (2020) 8:300. doi: 10.3390/vaccines8020300 32545304 PMC7350228

[33] Schaible UE Sturgill-Koszycki S Schlesinger PH Russell DG . Cytokine activation leads to acidification and increases maturation of Mycobacterium avium-containing phagosomes in murine macrophages. J Immunol. (1998) 160:1290–6. doi: 10.4049/jimmunol.160.3.1290 9570546

[34] MacMicking JD Taylor GA McKinney JD . Immune control of tuberculosis by IFN-gamma-inducible LRG-47. Science. (2003) 302:654–9. doi: 10.1126/science.1088063 14576437

[35] Deretic V Saitoh T Akira S . Autophagy in infection, inflammation and immunity. Nat Rev Immunol. (2013) 13:722–37. doi: 10.1038/nri3532 24064518 PMC5340150

[36] Zheng YT Shahnazari S Brech A Lamark T Johansen T Brumell JH . The adaptor protein p62/SQSTM1 targets invading bacteria to the autophagy pathway. J Immunol. (2009) 183:5909–16. doi: 10.4049/jimmunol.0900441 19812211

[37] Laplante M Sabatini DM . mTOR signaling in growth control and disease. Cell. (2012) 149:274–93. doi: 10.1016/j.cell.2012.03.017 22500797 PMC3331679

[38] Rubinsztein DC Codogno P Levine B . Autophagy modulation as a potential therapeutic target for diverse diseases. Nat Rev Drug Discov. (2012) 11:709–30. doi: 10.1038/nrd3802 22935804 PMC3518431

[39] Gutierrez MG Master SS Singh SB Taylor GA Colombo MI Deretic V . Autophagy is a defense mechanism inhibiting BCG and Mycobacterium tuberculosis survival in infected macrophages. Cell. (2004) 119:753–66. doi: 10.1016/j.cell.2004.11.038 15607973

[40] Fatima S Kumari A Das G Dwivedi VP . Tuberculosis vaccine: a journey from BCG to present. Life Sci. (2020) 252:117594. doi: 10.1016/j.lfs.2020.117594 32305522

[41] Liu PT Stenger S Li H Wenzel L Tan BH Krutzik SR . Toll-like receptor triggering of a vitamin D-mediated human antimicrobial response. Science. (2006) 311:1770–3. doi: 10.1126/science.1123933 16497887

[42] Singh SB Davis AS Taylor GA Deretic V . Human IRGM induces autophagy to eliminate intracellular mycobacteria. Science. (2006) 313:1438–41. doi: 10.1126/science.1129577 16888103

[43] Shin DM Jeon BY Lee HM Jin HS Yuk JM Song CH . Mycobacterium tuberculosis eis regulates autophagy, inflammation, and cell death through redox-dependent signaling. PloS Pathog. (2010) 6:e1001230. doi: 10.1371/journal.ppat.1001230 21187903 PMC3002989

[44] Diatlova A Linkova N Lavrova A Zinchenko Y Medvedev D Krasichkov A . Molecular markers of early immune response in tuberculosis: prospects of application in predictive medicine. Int J Mol Sci. (2023) 24:13261. doi: 10.3390/ijms241713261 37686061 PMC10487556

[45] Wolf AJ Desvignes L Linas B Banaiee N Tamura T Takatsu K . Initiation of the adaptive immune response to Mycobacterium tuberculosis depends on antigen production in the local lymph node, not the lungs. J Exp Med. (2008) 205:105–15. doi: 10.1084/jem.20071367 18158321 PMC2234384

[46] Geijtenbeek TBH Van Vliet SJ Koppel EA Sanchez-Hernandez M Vandenbroucke-Grauls CM Appelmelk B . Mycobacteria target DC-SIGN to suppress dendritic cell function. J Exp Med. (2003) 197:7–17. doi: 10.1084/jem.20021229 12515809 PMC2193797

[47] Lowe DM Redford PS Wilkinson RJ O'Garra A Martineau AR . Neutrophils in tuberculosis: friend or foe? Trends Immunol. (2012) 33:14–25. doi: 10.1016/j.it.2011.10.003 22094048

[48] Braian C Hogea V Stendahl O . Mycobacterium tuberculosis-induced neutrophil extracellular traps activate human macrophages. J Innate Immun. (2013) 5:591–602. doi: 10.1159/000348676 23635526 PMC6741595

[49] Berry MPR Graham CM McNab FW Xu Z Bloch SA Oni T . An interferon-inducible neutrophil-driven blood transcriptional signature in human tuberculosis. Nature. (2010) 466:973–7. doi: 10.1038/nature09247 20725040 PMC3492754

[50] Vankayalapati R Wizel B Weis SE Safi H Lakey DL Mandelboim O . The NKp46 receptor contributes to NK cell lysis of mononuclear phagocytes infected with an intracellular bacterium. J Immunol. (2002) 168:3451–7. doi: 10.4049/jimmunol.168.7.3451 11907104

[51] Vankayalapati R Garg A Porgador A Griffith DE Klucar P Safi H . Role of NK cell-activating receptors and their ligands in the lysis of mononuclear phagocytes infected with an intracellular bacterium. J Immunol. (2005) 175:4611–7. doi: 10.4049/jimmunol.175.7.4611 16177106

[52] Kleinnijenhuis J Quintin J Preijers F Joosten LA Ifrim DC Saeed S . Bacille Calmette-Guerin induces NOD2-dependent nonspecific protection from reinfection via epigenetic reprogramming of monocytes. Proc Natl Acad Sci USA. (2012) 109:17537–42. doi: 10.1073/pnas.1202870109 22988082 PMC3491454

[53] Shen Y Zhou D Qiu L Lai X Simon M Shen L . Adaptive immune response of Vgamma2Vdelta2+ T cells during mycobacterial infections. Science. (2002) 295:2255–8. doi: 10.1126/science.1068819 11910108 PMC2872146

[54] Lin PL Rutledge T Green AM Bigbee M Fuhrman C Klein E . CD4 T cell depletion exacerbates acute Mycobacterium tuberculosis while reactivation of latent infection is dependent on severity of tissue depletion in cynomolgus macaques. AIDS Res Hum Retroviruses. (2012) 28:1693–702. doi: 10.1089/aid.2012.0028 22480184 PMC3505050

[55] Flynn JL Chan J . Immunology of tuberculosis. Annu Rev Immunol. (2001) 19:93–129. doi: 10.1146/annurev.immunol.19.1.93 11244032

[56] Casanova JL Abel L . Genetic dissection of immunity to mycobacteria: the human model. Annu Rev Immunol. (2002) 20:581–620. doi: 10.1146/annurev.immunol.20.081501.125851 11861613

[57] Keane J Gershon S Wise RP Mirabile-Levens E Kasznica J Schwieterman WD . Tuberculosis associated with infliximab, a tumor necrosis factor α-neutralizing agent. N Engl J Med. (2001) 345:1098–104. doi: 10.1056/NEJMoa011110 11596589

[58] Khader SA Bell GK Pearl JE Fountain JJ Rangel-Moreno J Cilley GE . IL-23 and IL-17 in the establishment of protective pulmonary CD4+ T cell responses after vaccination and during Mycobacterium tuberculosis challenge. Nat Immunol. (2007) 8:369–77. doi: 10.1038/ni1449 17351619

[59] Lewinsohn DA Lewinsohn DM Scriba TJ . Polyfunctional CD4+ T cells as targets for tuberculosis vaccination. Front Immunol. (2017) 8:1262. doi: 10.3389/fimmu.2017.01262 29051764 PMC5633696

[60] Choi HG Kwon KW Choi S Back YW Park HS Kang SM . Antigen-specific IFN-γ/IL-17-co-producing CD4+ T cells are the determinants for protective efficacy of tuberculosis subunit vaccine. Vaccines (Basel). (2020) 8:300. doi: 10.3390/vaccines8020300 32545304 PMC7350228

[61] Stenger S Hanson DA Teitelbaum R Dewan P Niazi KR Froelich CJ . An antimicrobial activity of cytolytic T cells mediated by granulysin. Science. (1998) 282:121–5. doi: 10.1126/science.282.5386.121 9756476

[62] Behar SM Dascher CC Grusby MJ Wang CR Brenner MB . Susceptibility of mice deficient in CD1D or TAP1 to infection with Mycobacterium tuberculosis. J Exp Med. (1999) 189:1973–80. doi: 10.1084/jem.189.12.1973 10377193 PMC2192974

[63] Woodworth JS Aagaard CS Hansen PR Cassidy JP Agger EM Andersen P . CD4 T cell help prevents CD8 T cell exhaustion and promotes control of Mycobacterium tuberculosis infection. Cell Host Microbe. (2021) 29:1555–68. doi: 10.1016/j.chom.2021.09.005 34610294

[64] Van Rhijn I Moody DB . CD1 and mycobacterial lipids activate human T cells. Immunol Rev. (2015) 264:138–53. doi: 10.1111/imr.12253 25703557 PMC4339259

[65] Slight SR Rangel-Moreno J Gopal R Lin Y Fallert Junecko BA Mehra S . CXCR5+ T helper cells mediate protective immunity against tuberculosis. J Clin Invest. (2013) 123:712–26. doi: 10.1172/JCI65728 23281399 PMC3561804

[66] de Martino M Lodi L Galli L Chiappini E . Immune response to Mycobacterium tuberculosis: a narrative review. Front Pediatr. (2019) 7:350. doi: 10.3389/fped.2019.00350 31508399 PMC6718705

[67] Ramakrishnan L . Revisiting the role of the granuloma in tuberculosis. Nat Rev Immunol. (2012) 12:352–66. doi: 10.1038/nri3211 22517424

[68] Gideon HP Hughes TK Tzouanas CN Wadsworth M Tu AA Gierahn TM . Multimodal profiling of lung granulomas in macaques reveals cellular correlates of tuberculosis control. Immunity. (2022) 55:827–46. doi: 10.1016/j.immuni.2022.04.004 35483355 PMC9122264

[69] McCaffrey EF Donato M Keren L Chen Z Delmastro A Fitzpatrick MB . The immunoregulatory landscape of human tuberculosis granulomas. Nat Immunol. (2022) 23:318–29. doi: 10.1038/s41590-021-01121-x 35058616 PMC8810384

[70] Wherry EJ . T cell exhaustion. Nat Immunol. (2011) 12:492–9. doi: 10.1038/ni.2035 21739672

[71] Jayaraman P Jacques MK Zhu C Steblenko KM Stowell BL Madi A . TIM3 mediates T cell exhaustion during Mycobacterium tuberculosis infection. PloS Pathog. (2016) 12:e1005490. doi: 10.1371/journal.ppat.1005490 26967901 PMC4788425

[72] Moguche AO Musvosvi M Penn-Nicholson A Plumlee CR Mearns H Geldenhuys H . CD4 T cell dysfunction is associated with bacterial recrudescence during chronic tuberculosis. Nat Commun. (2025) 16:2581. doi: 10.1038/s41467-025-57819-1 40097414 PMC11914476

[73] Day CL Abrahams DA Behr L van Rooyen M Stone L de Kock M . PD-1 expression on Mycobacterium tuberculosis-specific CD4 T cells is associated with bacterial load in human tuberculosis. Front Immunol. (2018) 9:1995. doi: 10.3389/fimmu.2018.01995 30233588 PMC6127207

[74] Anand K Sahu G Burns E Ensor A Ensor J Pingali SR . Mycobacterial infections due to PD-1 and PD-L1 checkpoint inhibitors. ESMO Open. (2020) 5:e000866. doi: 10.1136/esmoopen-2020-000866 32817069 PMC7437685

[75] Lázár-Molnár E Chen B Sweeney KA Wang EJ Liu W Lin J . Programmed death-1 (PD-1)-deficient mice are extraordinarily sensitive to tuberculosis. Proc Natl Acad Sci USA. (2010) 107:13402–7. doi: 10.1073/pnas.1007394107 20624978 PMC2922129

[76] Barber DL Mayer-Barber KD Feng CG Sharpe AH Sher A . CD4 T cells promote rather than control tuberculosis in the absence of PD-1-mediated inhibition. J Immunol. (2011) 186:1598–607. doi: 10.4049/jimmunol.1003304 21172867 PMC4059388

[77] Tezera LB Bielecka MK Ogongo P Walker NF Ellis M Garay-Baquero DJ . Anti-PD-1 immunotherapy leads to tuberculosis reactivation via dysregulation of TNF-α. eLife. (2020) 9:e52668. doi: 10.7554/eLife.52668 32091388 PMC7058383

[78] Sharpe AH Freeman GJ . The B7-CD28 superfamily. Nat Rev Immunol. (2002) 2:116–26. doi: 10.1038/nri727 11910893

[79] Jayaraman P Jacques MK Zhu C Steblenko KM Stowell BL Madi A . TIM3 mediates T cell exhaustion during Mycobacterium tuberculosis infection. PloS Pathog. (2016) 12:e1005490. doi: 10.1371/journal.ppat.1005490 26967901 PMC4788425

[80] World Health Organization . Global Tuberculosis Report 2025: TB Research and Innovation. Geneva: World Health Organization (2025).

[81] Tait DR Hatherill M Van Der Meeren O Ginsberg AM Van Brakel E Salaun B . Final analysis of a trial of M72/AS01E vaccine to prevent tuberculosis. N Engl J Med. (2019) 381:2429–39. doi: 10.1056/NEJMoa1909953 31661198

[82] ClinicalTrials.gov . Phase 3 Study to Assess Efficacy and Safety of M72/AS01E-4 Mycobacterium Tuberculosis Vaccine in Adolescents and Adults (NCT06062238). (2023). United States: Gates Medical Research Institute

[83] Hatherill M Sebastian L Kagina BMN Bekker LG Hermosilla S Ginsberg AM . Safety and immunogenicity of investigational tuberculosis vaccine M72/AS01E-4 in people living with HIV in South Africa: an observer-blinded, randomised, controlled, phase 2 trial. Lancet HIV. (2025) 12:e456-65. doi: 10.1016/S2352-3018(25)00124-9 40614747 PMC12310912

[84] Luabeya AKK Kagina BM Tameris MD Geldenhuys H Hoff ST Shi Z . First-in-human trial of the post-exposure tuberculosis vaccine H56:IC31 in Mycobacterium tuberculosis infected and non-infected healthy adults. Vaccine. (2015) 33:4130–40. doi: 10.1016/j.vaccine.2015.06.051 26095509

[85] Coler RN Day TA Ellis R Piazza FM Beckmann AM Vergara J . The TLR-4 agonist adjuvant, GLA-SE, improves magnitude and quality of immune responses elicited by the ID93 tuberculosis vaccine. Tuberculosis (Edinb). (2018) 108:111–8. doi: 10.1016/j.tube.2017.11.011 30210819 PMC6123489

[86] Arbués A Aguilo JI Gonzalo-Asensio J Marinova D Uranga S Puentes E . Construction, characterization and preclinical evaluation of MTBVAC, the first live-attenuated M. tuberculosis-based vaccine to enter clinical trials. Vaccine. (2013) 31:4867–73. doi: 10.1016/j.vaccine.2013.07.051 23965219

[87] Tameris M Mearns H Penn-Nicholson A Gregg Y Bilek N Mabwe S . Live-attenuated Mycobacterium tuberculosis vaccine MTBVAC versus BCG in adults and neonates: a randomised controlled, double-blind dose-escalation trial. Lancet Respir Med. (2019) 7:757–70. doi: 10.1016/S2213-2600(19)30251-6 31416768

[88] Nieuwenhuizen NE Kulkarni PS Shaligram U Cotton MF Rentsch CA Eisele B . The recombinant Bacille Calmette-Guérin vaccine VPM1002: ready for clinical efficacy testing. Front Immunol. (2017) 8:1147. doi: 10.3389/fimmu.2017.01147 28974949 PMC5610719

[89] Tameris MD Hatherill M Landry BS Scriba TJ Snowden MA Lockhart S . Safety and efficacy of MVA85A, a new tuberculosis vaccine, in infants previously vaccinated with BCG: a randomised, placebo-controlled phase 2b trial. Lancet. (2013) 381:1021–8. doi: 10.1016/S0140-6736(13)60177-4 23391465 PMC5424647

[90] Smaill F Jeyanathan M Smieja M Medina MF Thanthrige-Don N Zganiacz A . A human type 5 adenovirus-based tuberculosis vaccine induces robust T cell responses in humans despite preexisting anti-adenovirus immunity. Sci Transl Med. (2013) 5:205ra134. doi: 10.1126/scitranslmed.3006843 24089406

[91] ClinicalTrials.gov . TB/FLU-05E Phase I Clinical Trial (NCT05945498). (2023). Russia: Research Institute of Influenza

[92] Vogel AB Kanevsky I Che Y Swanson KA Muik A Vormehr M . mRNA-based tuberculosis vaccines BNT164a1 and BNT164b1 are immunogenic, well-tolerated and efficacious in rodent models. In: Biorxiv. Mainz, Germany: BioNTech SE (2025). doi: 10.1101/2025.09.30.679428 PMC13414557 42286358

[93] ClinicalTrials.gov . BNT164a1 and BNT164b1 in Tuberculosis – Phase Ia Clinical Trial (NCT05537038). (2022).

[94] Vogel AB Kanevsky I Che Y Swanson KA Muik A Vormehr M . mRNA-based tuberculosis vaccines BNT164a1 and BNT164b1. In: Biorxiv. Mainz, Germany: BioNTech SE (2025). doi: 10.1101/2025.09.30.679428

[95] Yang XY Chen QF Li YP Wu SM . Mycobacterium vaccae as adjuvant therapy to anti-tuberculosis chemotherapy in never-treated tuberculosis patients: a meta-analysis. PloS One. (2011) 6:e23826. doi: 10.1371/journal.pone.0023826 21909406 PMC3167806

[96] Nell AS D'lom E Bouic P Sabaté M Bosser R Picas J . Safety, tolerability, and immunogenicity of the novel antituberculous vaccine RUTI: randomized, placebo-controlled phase II clinical trial in patients with latent tuberculosis infection. PloS One. (2014) 9:e89612. doi: 10.1371/journal.pone.0089612 24586912 PMC3935928

[97] von Reyn CF Mtei L Arbeit RD Waddell R Cole B Mackenzie T . Prevention of tuberculosis in Bacille Calmette-Guérin-primed, HIV-infected adults boosted with an inactivated whole-cell mycobacterial vaccine. AIDS. (2010) 24:675–85. doi: 10.1097/QAD.0b013e3283350f1b 20118767 PMC10525041

[98] Singhal A Jie L Kumar P Hong GS Leow MK Paleja B . Metformin as adjunct antituberculosis therapy. Sci Transl Med. (2014) 6:263ra159. doi: 10.1126/scitranslmed.3009885 25411472

[99] Oglesby W Kara AM Granados H Cervantes JL . Metformin in tuberculosis: beyond control of hyperglycemia. Infection. (2019) 47:697–702. doi: 10.1007/s15010-019-01322-5 31119504

[100] Singhal A Jie L Kumar P Hong GS Leow MK Paleja B . Metformin as adjunct antituberculosis therapy. Sci Transl Med. (2014) 6:263ra159. doi: 10.1126/scitranslmed.3009885 25411472

[101] Pan SW Yen YF Kou YR Chuang PH Su VY Feng JY . The risk of TB in patients with type 2 diabetes initiating metformin vs sulfonylurea treatment. Chest. (2018) 153:1347–57. doi: 10.1016/j.chest.2017.11.040 29253553

[102] Ma Y Pang Y Shu W Liu YH Ge QP Du J . Metformin reduces the relapse rate of tuberculosis patients with diabetes mellitus: experiences from 3-year follow-up. Eur J Clin Microbiol Infect Dis. (2018) 37:1259–63. doi: 10.1007/s10096-018-3242-6 29679254

[103] Padmapriydarsini C Mamulwar M Mohan A Shanmugam P Gomathy NS Mane A . Randomized trial of metformin with anti-tuberculosis drugs for early sputum conversion in adults with pulmonary tuberculosis. Clin Infect Dis. (2022) 75:425–34. doi: 10.1093/cid/ciab964 34849651 PMC9427151

[104] Yuk JM Shin DM Lee HM Yang CS Jin HS Kim KK . Vitamin D3 induces autophagy in human monocytes/macrophages via cathelicidin. Cell Host Microbe. (2009) 6:231–43. doi: 10.1016/j.chom.2009.08.004 19748465

[105] Papagni R Pellegrino C Di Gennaro F Patti G Ricciardi A Novara R . Impact of vitamin D in prophylaxis and treatment in tuberculosis patients. Int J Mol Sci. (2022) 23:3860. doi: 10.3390/ijms23073860 35409219 PMC8999210

[106] Nnoaham KE Clarke A . Low serum vitamin D levels and tuberculosis: a systematic review and meta-analysis. Int J Epidemiol. (2008) 37:113–9. doi: 10.1093/ije/dym247 18245055

[107] Salahuddin N Ali F Hasan Z Rao N Aqeel M Mahmood F . Vitamin D accelerates clinical recovery from tuberculosis: results of the SUCCINCT Study (Supplementary Cholecalciferol in recovery from tuberculosis). A randomized, placebo-controlled, clinical trial of vitamin D supplementation in patients with pulmonary tuberculosis. BMC Infect Dis. (2013) 13:22. doi: 10.1186/1471-2334-13-22 23331510 PMC3556334

[108] Tukvadze N Sanikidze E Kipiani M Hebbar G Easley KA Shenvi N . High-dose vitamin D3 in adults with pulmonary tuberculosis: a double-blind randomized controlled trial. Am J Clin Nutr. (2015) 102:1059–69. doi: 10.3945/ajcn.115.113886 26399865 PMC4625591

[109] Martineau AR Timms PM Bothamley GH Hanifa Y Islam K Claxton AP . High-dose vitamin D3 during intensive-phase antimicrobial treatment of pulmonary tuberculosis: a double-blind randomised controlled trial. Lancet. (2011) 377:242–50. doi: 10.1016/S0140-6736(10)61889-2 21215445 PMC4176755

[110] Parihar SP Guler R Khutlang R Lang DM Hurdayal R Mhlanga MM . Statin therapy reduces the Mycobacterium tuberculosis burden in human macrophages and in mice by enhancing autophagy and phagosome maturation. J Infect Dis. (2014) 209:754–63. doi: 10.1093/infdis/jit550 24133190

[111] Pandey AK Sassetti CM . Mycobacterial persistence requires the utilization of host cholesterol. Proc Natl Acad Sci USA. (2008) 105:4376–80. doi: 10.1073/pnas.0711159105 18334639 PMC2393810

[112] Dutta NK Bruiners N Pinn ML Zimmerman MD Prideaux B Dartois V . Statin adjunctive therapy shortens the duration of TB treatment in mice. J Antimicrob Chemother. (2016) 71:1570–7. doi: 10.1093/jac/dkw014 26903278 PMC5007636

[113] Niemi M Backman JT Fromm MF Neuvonen PJ Kivistö KT . Pharmacokinetic interactions with rifampicin: clinical relevance. Clin Pharmacokinet. (2003) 42:819–50. doi: 10.2165/00003088-200342090-00003 12882588

[114] Elkington P Shiomi T Breen R Nuttall RK Ugarte-Gil CA Walker NF . MMP-1 drives immunopathology in human tuberculosis and transgenic mice. J Clin Invest. (2011) 121:1827–33. doi: 10.1172/JCI45666 21519144 PMC3083790

[115] Walker NF Clark SO Oni T Andreu N Tezera L Singh S . Doxycycline and HIV infection suppress tuberculosis-induced matrix metalloproteinases. Am J Respir Crit Care Med. (2012) 185:989–97. doi: 10.1164/rccm.201110-1769OC 22345579 PMC3359940

[116] Andrade BB Pavan Kumar N Amaral EP Riteau N Mayer-Barber KD Tosh KW . Heme oxygenase-1 regulation of matrix metalloproteinase-1 expression underlies distinct disease profiles in tuberculosis. J Immunol. (2015) 195:2763–73. doi: 10.4049/jimmunol.1500942 26268658 PMC4561190

[117] Vilaplana C Marzo E Tapia G Diaz J Garcia V Cardona PJ . Ibuprofen therapy resulted in significantly decreased tissue bacillary loads and increased survival in a new murine experimental model of active tuberculosis. J Infect Dis. (2013) 208:199–202. doi: 10.1093/infdis/jit152 23564636

[118] Chen M Divangahi M Gan H Shin DS Hong S Lee DM . Lipid mediators in innate immunity against tuberculosis: opposing roles of PGE2 and LXA4 in the induction of macrophage death. J Exp Med. (2008) 205:2791–801. doi: 10.1084/jem.20080767 18955568 PMC2585850

[119] Prasad K Singh MB Ryan H . Corticosteroids for managing tuberculous meningitis. Cochrane Database Syst Rev. (2016) 4:CD002244. doi: 10.1002/14651858.CD002244.pub4 27121755 PMC4916936

[120] Condos R Rom WN Schluger NW . Treatment of multidrug-resistant pulmonary tuberculosis with interferon-gamma via aerosol. Lancet. (1997) 349:1513–5. doi: 10.1016/S0140-6736(96)12273-X 9167461

[121] Johnson JL Ssekasanvu E Okwera A Mayanja H Hirsch CS Nakibali JG . Randomized trial of adjunctive interleukin-2 in adults with pulmonary tuberculosis. Am J Respir Crit Care Med. (2003) 168:185–91. doi: 10.1164/rccm.200211-1359OC 12702550

[122] Pedral-Sampaio DB Netto EM Brites C Bandeira AC Guerra C Barberin MG . Use of Rhu-GM-CSF in pulmonary tuberculosis patients: results of a randomized clinical trial. Braz J Infect Dis. (2003) 7:245–52. doi: 10.1590/s1413-86702003000400004 14533985

[123] Rivas-Santiago B Hernandez-Pando R Carranza C Juarez E Contreras JL Aguilar-Leon D . Expression of cathelicidin LL-37 during Mycobacterium tuberculosis infection in human alveolar macrophages, monocytes, neutrophils, and epithelial cells. Infect Immun. (2008) 76:935–41. doi: 10.1128/IAI.01218-07 18160480 PMC2258801

[124] Gao X Feng J Wei L Dong P Chen J Zhang L . Defensins: a novel weapon against Mycobacterium tuberculosis? Int Immunopharmacol. (2024) 127:111383. doi: 10.1016/j.intimp.2023.111383 38118315

[125] Singh DK Tousif S Bhaskar A Devi A Negi K Moitra B . Luteolin as a potential host-directed immunotherapy adjunct to isoniazid treatment of tuberculosis. PloS Pathog. (2021) 17:e1009805. doi: 10.1371/journal.ppat.1009805 34415976 PMC8409628

[126] McKell MC Crowther RR Schmidt SM Robillard MC Cantrell R Lehn MA . Promotion of anti-tuberculosis macrophage activity by L-arginine in the absence of nitric oxide. Front Immunol. (2021) 12:653571. doi: 10.3389/fimmu.2021.653571 34054815 PMC8160513

[127] Fatima S Bhaskar A Dwivedi VP . Repurposing immunomodulatory drugs to combat tuberculosis. Front Immunol. (2021) 12:645485. doi: 10.3389/fimmu.2021.645485 33927718 PMC8076598

[128] Gallegos AM Pamer EG Glickman MS . Delayed protection by ESAT-6-specific effector CD4+ T cells after airborne M. tuberculosis infection. J Exp Med. (2008) 205:2359–68. doi: 10.1084/jem.20080353 18779346 PMC2556792

[129] Zhuang L Yang L Li L Ye Z Gong W . Mycobacterium tuberculosis: immune response, biomarkers, and therapeutic intervention. MedComm. (2024) 5:e419. doi: 10.1002/mco2.419 38188605 PMC10771061

[130] Guyot-Revol V Innes JA Hackforth S Hinks T Lalvani A . Regulatory T cells are expanded in blood and disease sites in patients with tuberculosis. Am J Respir Crit Care Med. (2006) 173:803–10. doi: 10.1164/rccm.200508-1294OC 16339919

[131] Skrahin A Ahmed RK Ferrara G Rane L Poiret T Isaikina Y . Autologous mesenchymal stromal cell infusion as adjunct treatment in patients with multidrug and extensively drug-resistant tuberculosis: an open-label phase 1 safety trial. Lancet Respir Med. (2014) 2:108–22. doi: 10.1016/S2213-2600(13)70234-0 24503266

[132] World Health Organization . Global Tuberculosis Report 2024. Geneva: World Health Organization (2024).

[133] Conradie F Diacon AH Ngubane N Howell P Everitt D Crook AM . Treatment of highly drug-resistant pulmonary tuberculosis. N Engl J Med. (2020) 382:893–902. doi: 10.1056/NEJMoa1901814 32130813 PMC6955640

[134] Scriba TJ Netea MG Ginsberg AM . Key recent advances in TB vaccine development and understanding of protective immune responses against Mycobacterium tuberculosis. Semin Immunol. (2020) 50:101431. doi: 10.1016/j.smim.2020.101431 33279383 PMC7786643

[135] Andersen P Scriba TJ . Moving tuberculosis vaccines from theory to practice. Nat Rev Immunol. (2019) 19:550–62. doi: 10.1038/s41577-019-0174-z 31114037

[136] Ogongo P Tezera LB Ardain A Nhamoyebonde S Ramsuran D Singh A . Tissue-resident-like CD4+ T cells secreting IL-17 control Mycobacterium tuberculosis in the human lung. J Clin Invest. (2021) 131:e142014. doi: 10.1172/JCI142014 33848273 PMC8121523

[137] Krausgruber T Fortelny N Fife-Gernedl V Senekowitsch M Schuster LC Lercher A . Structural cells are key regulators of organ-specific immune responses. Nature. (2020) 583:296–302. doi: 10.1038/s41586-020-2424-4 32612232 PMC7610345

[138] Schäferhoff M Zimmerman A Diab MM Mao W Chowdhary V Gill D . Investing in late-stage clinical development of new tuberculosis vaccines. Lancet Infect Dis. (2023) 23:e165–70. doi: 10.1016/S2214-109X(22)00206-6 38561009

[139] World Health Organization . Tb Vaccine Accelerator Council Progress Update. Geneva: World Health Organization (2025).

[140] Costa A Pinheiro M Magalhães J Ribeiro R Seabra V Reis S . The formulation of nanomedicines for treating tuberculosis. Adv Drug Delivery Rev. (2016) 102:102–15. doi: 10.1016/j.addr.2016.04.012 27108703

[141] Bhargava A Bhargava M Meher A Benedetti A Velayutham B Teja GS . Nutritional supplementation to prevent tuberculosis incidence in household contacts of patients with pulmonary tuberculosis in India (RATIONS): a field-based, open-label, cluster-randomised, controlled trial. Lancet. (2023) 402:627–40. doi: 10.1016/S0140-6736(23)01231-X 37567200

[142] Bhargava A Bhargava M Meher A Teja GS Velayutham B Watson B . Nutritional support for adult patients with microbiologically confirmed pulmonary tuberculosis: outcomes in a programmatic cohort nested within the RATIONS trial in Jharkhand, India. Lancet Glob Health. (2023) 11:e1402–11. doi: 10.1016/S2214-109X(23)00324-8 37567210

[143] Sinha P Lönnroth K Bhargava A Heysell SK Pai M Christopher DJ . Food for thought: addressing undernutrition to end tuberculosis. Lancet Infect Dis. (2021) 21:e318–25. doi: 10.1016/S1473-3099(20)30792-1 33770535 PMC8458477

[144] Bhargava A Bhargava M Benedetti A Kurpad A Menzies D . Attributable is preventable: corrected and revised estimates of population attributable fraction of TB related to undernutrition in 30 high TB burden countries. J Clin Tuberc Other Mycobact Dis. (2022) 27:100309. doi: 10.1016/j.jctube.2022.100309 35308808 PMC8924683

